# Lipopolysaccharide exposure during late embryogenesis triggers and drives Alzheimer‐like behavioral and neuropathological changes in CD‐1 mice

**DOI:** 10.1002/brb3.1546

**Published:** 2020-01-30

**Authors:** Fang Wang, Zhe‐Zhe Zhang, Lei Cao, Qi‐Gang Yang, Qing‐Fang Lu, Gui‐Hai Chen

**Affiliations:** ^1^ Department of Neurology the First Affiliated Hospital of Anhui Medical University Hefei China; ^2^ Department of Neurology the Second Affiliated Hospital of Anhui Medical University Hefei China; ^3^ Department of Critical Care Medicine the First Affiliated Hospital of Anhui Medical University Hefei China; ^4^ Department of Mental Psychology the Taihe County Chinese Medicine Hospital Fuyang China; ^5^ Department of Neurology (Sleep Disorders) the Affiliated Chaohu Hospital of Anhui Medical University Hefei China

**Keywords:** aging, Alzheimer's disease, lipopolysaccharide, memory, mice

## Abstract

**Introduction:**

Infections could contribute to Alzheimer's disease (AD) neuropathology in human. However, experimental evidence for a causal relationship between infections during the prenatal phase and the onset of AD is lacking.

**Methods:**

CD‐1 mothers were intraperitoneally received lipopolysaccharide (LPS) with two doses (25 and 50 μg/kg) or normal saline every day during gestational days 15–17. A battery of behavioral tasks was used to assess the species‐typical behavior, sensorimotor capacity, anxiety, locomotor activity, recognition memory, and spatial learning and memory in 1‐, 6‐, 12‐, 18‐, and 22‐month‐old offspring mice. An immunohistochemical technology was performed to detect neuropathological indicators consisting of amyloid‐β (Aβ), phosphorylated tau (p‐tau), and glial fibrillary acidic protein (GFAP) in the hippocampus.

**Results:**

Compared to the same‐aged controls, LPS‐treated offspring had similar behavioral abilities and the levels of Aβ42, p‐tau, and GFAP at 1 and 6 months old. From 12 months onward, LPS‐treated offspring gradually showed decreased species‐typical behavior, sensorimotor ability, locomotor activity, recognition memory, and spatial learning and memory, and increased anxieties and the levels of Aβ42, p‐tau, and GFAP relative to the same‐aged controls. Moreover, this damage effect (especially cognitive decline) persistently progressed onwards. The changes in these neuropathological indicators significantly correlated with impaired spatial learning and memory.

**Conclusions:**

Prenatal exposure to low doses of LPS caused AD‐related features including behavioral and neuropathological changes from midlife to senectitude.

## Graphical abstract

Prenatal exposure to low doses of lipopolysaccharide (LPS) caused Alzheimer's disease (AD)‐like features. In later life, LPS‐treated mice showed increased expression of Aβ42, p‐tau, and glial fibrillary acidic protein (GFAP) and these neuropathological indicators correlated with impaired spatial cognition.

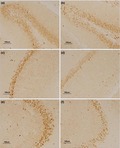

## 1 INTRODUCTION

Alzheimer's disease (AD) is the most prevalent form of age‐related dementia, which is characterized by a wide range of symptoms, such as gradually degenerating cognitive abilities, behavioral disorders, personality changes, and motor and sensory deficits (van Wijngaarden, Hadoux, Alwan, Keel, & Dirani, 2017). The major neuropathological hallmarks of AD include neuronal and synaptic loss, and proteinaceous aggregates in the form of senile plaques, composed of amyloid‐β (Aβ) peptides as well as neurofibrillary tangles, consisting of hyperphosphorylated tau (p‐tau) in the brain (Overk & Masliah, 2014; Ramirez et al., 2017). Moreover, neuroinflammation and astrogliosis proliferation, recruitment, and activation are commonly associated with AD pathology (Steardo et al., 2015). The factors and molecular mechanisms that affect the pathogenesis of late‐onset AD remain largely unknown, although it is widely accepted that this disorder has a complex etiology involving both genetic (minor risk genes) and environmental factors (Castellani, Rolston, & Smith, 2010).

Accumulating evidence indicates the possible association between various microbial infections and AD onset and progression (Ashraf et al., 2019). Preclinical research suggests maternal immune activation (mIA) might precipitate the development of AD (Knuesel et al., 2014). For instance, polyriboinosinic–polyribocytidilic acid (poly I:C)‐induced mIA during late gestation predisposes wild‐type mice to develop AD‐like neuropathology throughout aging (Meghraj et al., 2017). These mice display serious spatial learning and memory impairments, chronic elevation of inflammatory cytokines, increased levels of hippocampal amyloid precursor protein (APP) with its proteolytic fragments, and altered Tau phosphorylation in old age (Krstic et al., 2012; Meghraj et al., 2017). However, there is missing experimental evidence to support an early and potentially causality for maternal systemic infections in the progeny etiology of sporadic AD.

Bacterial infections have a high prevalence in women of reproductive age. Increasing evidence indicates that modifications of the “in utero” environment due to maternal bacterial infection can result in cognitive and behavioral disorders in pre‐ or adult offspring, such as impairments in spatial learning and memory (Batinic et al., 2016; Chlodzinska, Gajerska, Bartkowska, Turlejski, & Djavadian, 2011; Glass, Norton, Fox, & Kusnecov, 2019; Simões et al., 2018) and object recognition (Glass et al., 2019; Wischhof, Irrsack, Osorio, & Koch, 2015), increased locomotor activity (Batinic et al., 2016; Glass et al., 2019) and anxiety (Enayati et al., 2012; Glass et al., 2019; Hsueh et al., 2017; Penteado et al., 2014) and decreased prepulse inhibition of acoustic startle (Fortier, Luheshi, & Boksa, 2007; Glass et al., 2019; Wischhof et al., 2015) and social behaviors (Glass et al., 2019; Hsueh et al., 2017). Lipopolysaccharide (LPS) injection in the pregnancy is a widely accepted mouse model of maternal bacterial infection. A limited number of studies have investigated the age‐related cognitive and behavioral consequences in these offspring, particularly from midlife to senectitude. Pregnant Sprague Dawley rats treated intraperitoneal injection (i.p.) with LPS 0.79 mg/kg at gestational days (gd) 8, 10, and 12 showed distinct learning and memory decline in their offspring at the ages of 10 and 20 months but not 3 months (Hao, Hao, Li, & Li, 2010). Our previous studies indicated that pregnant CD‐1 mice intraperitoneally received 50 μg/kg LPS during gd 15–17 accelerated age‐related learning and memory impairment and species‐typical behaviors in middle‐aged offspring (Chen et al., 2011; Li, Cao, et al., 2016; Li, Wang, et al., 2016). Moreover, this LPS effect on learning and memory deficit was also observed in old‐aged offspring mice and thus was even a lower‐dose injection of LPS (25 μg/kg; Li, Cao, et al., 2016; Li, Wang, et al., 2016).

Besides behavioral and cognitive dysfunctions, maternal infection insult by LPS can lead to certain changes in hippocampal morphology and neurochemistry in offspring, such as neuron loss, altered synaptic transmission, reduced hippocampal neurogenesis, decreased expression of synaptophysin, and increased expression of GFAP in the hippocampal CA1 region (Boksa, 2010; Graciarena, Depino, & Pitossi, 2010; Hao et al., 2010; Lowe, Luheshi, & Williams, 2008). A recent investigation by our group showed that maternal inflammatory insult by LPS administration during pregnancy worsened the age‐related hippocampal neurobiological indicators (decreased H4K8ac, H3K9ac, and Stx‐1 and increased Syt‐1) in the offspring of CD‐1 mice from midlife (12 months old) to the twilight years (22 months old; Li, Cao, et al., 2016; Li, Wang, et al., 2016). However, these previous studies did not comprehensively assess cognitive and behavioral functions in these mice suffered with LPS during late embryogenesis at different age especially old age, nor did they detect AD‐related pathophysiology in the hippocampus.

Based on the aforementioned background, the current investigation was carried out to explore whether maternal exposure to LPS exacerbates: (a) The age‐related behavioral changes assessed by a battery of behavioral tasks in the offspring CD‐1 mice from adolescence and twilight years; (b) the age‐related changes of Aβ, p‐tau, and GFAP in the hippocampus quantified using immunohistochemical staining. In addition, the senile plaques and neurofibrillary tangles were detected by Congo red and Bielschowsky silver staining, and the correlations between spatial learning and memory and measured neuropathological indicators were also analyzed. Ultimately, we evaluated whether the behavioral and neuropathological characteristics in the brain were in accordance with those in AD.

## 2 MATERIALS AND METHODS

### 2.1 Animals and general procedures

Seven‐ to eight‐week‐old CD‐1 mice (40 males and 80 females) were bought from Vital River Laboratory Animal Technology Co. Ltd. These mice were fed in a controlled temperature (20–25°C) and humidity (50 ± 5%) environment with 12 hr light–dark cycle. After they acclimated for 1 week, the males and females (1:2) were paired into breeders. The emergence of a vaginal plug was considered gd 0. All pregnant mice were injected intraperitoneally with LPS (50 or 25 μg/kg, serotype 0127: B8, L3129; Sigma) or normal saline daily during gd15–17. Their offspring mice were, respectively, designated as higher‐dose LPS (H‐LPS), lower‐dose LPS (L‐LPS), and control (CON) groups. On postnatal day 21, these mice were separated from their mothers and siblings, and 4–5 mice of the same sex were housed in the same cage. During all tasks and their lifetime, they received a standard rodent diet and free tap water. We carried out all animal procedures according to the recommendations of the National Institutes of Health (NIH) Guide for the Care and Use of Laboratory Animals, and the Center for Laboratory Animal Sciences at Anhui Medical University.

One male and one female offspring mouse per litter (eight males and eight females) were measured daily for body weight during 21–30 days and once at intervals of 2 months from 2 to 22 months, and then, they were sacrificed. One male and one female offspring mouse per litter (eight males and eight females) were assessed for complete behaviors at 1, 6, 12, 18, and 22 months old. Given the limitation of behavioral tasks in a longitudinal study, for example, retest effects, the animals were not retested and sacrificed at different age of detection in the study. With the exception of nesting task, each task was conducted during the light phase. The battery of behavioral tasks consisted of species‐typical behavior (nesting), sensorimotor‐based task (beam walk), anxiety‐based tasks (open field and elevated plus maze), locomotor activity (open field), and cognitive tasks (object location recognition [OLR] and radial six‐arm water maze [RAWM]). They were carried out in the following order: nesting, open field, beam walking, elevated plus maze, OLR, and RAWM. In order to adapt the environment, all tasks were conducted in the feeding room.

### 2.2 Behavioral test

The behavioral experiments including nesting, open field, beam walking, elevated plus maze, OLR, and RAWM were conducted according to our previous studies (Chen et al., 2011; Li, Cao, et al., 2016; Li, Wang, et al., 2016; Tong et al., 2015).

### 2.3 Tissue preparation

After completing the behavioral experiment, the mice were anesthetized with halothane and sacrificed. Brains were rapidly removed and bisected in the mid‐sagittal plane, fixated in 4% paraformaldehyde at 4°C for 12 hr, and paraffin‐embedded for immunohistochemistry. Coronal sections were cut at a 6 μm thickness from tissue paraffin blocks using a microtome.

### 2.4 Congo red staining and Bielschowsky silver staining

#### 2.4.1 Congo red staining

Tissue slides were deparaffinized in xylenes and rehydrated in graded alcohols, and then, they were washed in distilled water three times. First, the sections were stained with Congo red for 20 min at room temperature, and alkaline alcohol was used to differentiate slides for seconds before they were rinsed in running water for 5 min. The sections were immersed in hematoxylin for 2 min and then rinsed in tap water until it turned blue. Finally, the sections were eliminated in xylene and then covered with neutral gum.

#### 2.4.2 Modified Bielschowsky silver staining

After tissue slides were deparaffinized in xylenes and rehydrated in graded alcohols, they were washed in distilled water three times. Firstly, they were immersed in 3% argent nitrate solution for 35 min at 37°C in the dark and were rinsed in distilled water for 3 min. 10% formaldehyde was used to deoxidize the staining until the slide turned into a pale‐yellow color. These slides were washed in distilled water for 3 min and stained using ammoniacal silver solution for 30 s. Then, these slides were rotated several times until the yellow dye became stable. The sections were mixed colors by gold chloride solution for 3 min before being washed in distilled water for 3 min. The sections were fixed in 5% sodium thiosulfate for 5 min and washed in distilled water for 3 min. Finally, the sections were eliminated in xylene and then covered with neutral gum.

### 2.5 Immunohistochemical staining

The strept–avidin–biotin–peroxidase complex (SABC) method was performed as described in our previous studies (Li, Cao, et al., 2016; Li, Wang, et al., 2016; Tong et al., 2015). The main difference is that primary antibodies including rabbit monoclonal anti‐Aβ_42_ (1:300) and polyclonal anti‐p‐tauser404 (1:500) and GFAP (1:500) were purchased from the Abcam and Dako.

### 2.6 Statistical analysis

The results were expressed as mean ± standard deviation for the parametric data or median (25th/75th quartile) for the nonparametric data. For the data from RAWM task and body weight, analysis was performed using a repeated‐measures analysis of variance (rm‐ANOVA) with Fisher's least‐significant difference test for post hoc analysis to compare the results among the different groups. The parametric data were analyzed using a two‐way ANOVA with group (treatment) and sex as independent variables. For the nonparametric data, the Kruskal–Wallis *H* test was used. Pearson's correlation test was conducted to analyze the correlations between the relative levels of hippocampal proteins and RAWM performance. Significance was assumed when *p* < .05. The statistical software SPSS 13.0 was used for the statistical analysis.

## 3 RESULTS

### 3.1 Body weight

The body weight results are shown in Figure S1. The rm‐ANOVAs showed that body weight was similar among LPS‐treated and control mice during 21–30 days and 2–22 months for all mice combined (*p*s > .05). The males had more body weight than the females [*F*_(1, 64)_ = 27.827, *p* < .001].

### 3.2 Behaviors in the 1‐ and 6‐month‐old mice

There is insignificant LPS treatment effect on the parameters of the nesting, beam walking, open field, elevated plus maze, OLR, and RAWM tests for the combined and separated sexes (*p*s > .05; see Table S1 and Figures S2 and S3).

### 3.3 Behaviors in the 12‐month‐old mice

#### 3.3.1 Nesting and Beam walking

There were no major differences in the performance of the nesting and beam walking tests among the LPS mice and the control ones for the combined and separated sexes (*p*s > .05, Table 1).

**Table 1:** The behavioral results of different‐treated CD‐1 mice at the age of 12, 18, and 22 months

Tasks	Index	Ages	H‐LPS group			L‐LPS group			Controls		
			All mice	Males	Females	All mice	Males	Females	All mice	Males	Females
Nesting	Scores	12‐month	3.0 (2.0/3.0)	3.0 (2.25/3.0)	2.5 (1.25/3.75)	2.0 (1.25/3.75)	3.0 (2.25/4.0)	2.0 (1.0/2.0)	2.0 (1.0/3.0)	2.0 (1.0/3.0)	2.5 (0.5/3.75)
		18‐month	1.0 (1.0/2.75)*	1.0 (0.25/2.5)*	2.0 (1.0/2.75)*	3.0 (2.0/3.75)	2.5 (2.0/4.0)	3.0 (2.0/3.0)	3.0 (2.0/4.0)	3.0 (2.0/4.0)	3.0 (2.0/4.0)
		22‐month	1.5 (0.25/2.0)*	2.0 (0.25/2.0)*	1.0 (0/2.0)*	2.0 (1.0/3.0)*	1.5 (0.25/3.75)*	2.0 (1.0/2.75)	3.0 (2.0/4.0)	3.0 (2.0/4.0)	2.5 (2.0/3.75)
Beam walking	Time (s)	12‐month	60.0 (44.58/60.0)	60 (23.33/60.0)	60.0 (50.0/60.0)	60 (41.17/60.0)	50.8 (35.42/60.0)	60.0 (45.25/60.0)	60.0 (42.25/60.0)	53.8 (46.25/60.0)	54.5 (39.0/60.0)
		18‐month	34.7 (27.8/51.5)*	30 (7.0/48.5)*	42.3 (31.4/60.0)	51.8 (44.0/60.0)	51.8 (44.0/60.0)	51.2 (33.25/60.0)	60.0 (48.42/60.0)	60.0 (48.75/60.0)	60 (48.42/60.0)
		22‐month	27.0 (19.78/39.3)*	27.0 (17.33/35.5)*	27.7 (21.5/46.6)*	38.5 (11.7/58.5)*	30.7 (11.25/48.5)*	47.7 (13.3/60.0)	56.3 (44.8/60.0)	58.3 (45.5/60.0)	55.0 (44.83/60.0)
Open field	Peripheral time (s)	12‐month	273.9 ± 5.21	266.0 ± 7.37	281.7 ± 7.37	270.3 ± 5.21	266.4 ± 7.37	274.1 ± 7.37	263.8 ± 5.21	260.3 ± 7.37	266.1 ± 7.37
		18‐month	270.1 ± 6.28* ^,^ †	278.6 ± 8.88*	261.5 ± 8.88* ^,^ †	242.3 ± 6.28	256.5 ± 8.88	227.7 ± 8.88	230.2 ± 6.28	240.3 ± 8.88	220.1 ± 8.88
		22‐month	264.7 ± 6.40*	259.5 ± 9.05	269.8 ± 9.05*	252.9 ± 6.40	257.7 ± 9.05	248.1 ± 9.05	239.4 ± 6.40	240.0 ± 9.05	238.9 ± 9.05
	Squares crossed	12‐month	104.6 ± 13.9*	116.5 ± 12.66	92.6 ± 12.66	118.4 ± 13.9	105.9 ± 12.66	130.8 ± 12.66	153.7 ± 13.9	130.9 ± 12.66	176.5 ± 12.66
		18‐month	147.6 ± 9.46*	138.9 ± 11.51	156.4 ± 11.51*	176.9 ± 9.46	146.8 ± 11.51	197.6 ± 11.51	214.3 ± 9.46	177.9 ± 11.51	236.5 ± 11.51
		22‐month	118.2 ± 7.91*	112.8 ± 11.18*	123.6 ± 11.18	134.3 ± 7.91	124.8 ± 11.18	143.9 ± 11.18	143.1 ± 7.91	142.3 ± 11.18	147.3 ± 11.18
Elevated plus maze	Number of entries	12‐month	1.0 (0/2.75)	1.0 (0/2.75)	1.0 (0.25/2.75)	1.0 (0.25/2.0)	1.0 (0/2.0)	1.0 (1.0/2.75)	2.0 (1.0/4.5)	2.0 (1.0/5.75)	2.0 (0.25/2.75)
		18‐month	1.0 (0.0/3.0)	1.0 (0.0/2.5)	1.0 (0.0/3.0)	1.5 (1.0/4.0)	2.0 (1.0/4.0)	1.5 (1.0/4.0)	2.0 (1.0/3.0)	1.5 (0.25/2.75)	2.0 (2.0/3.0)
		22‐month	2.0 (0/3.75)*	3.5 (0/4.0)	1.5 (0.25/2.0)*	3.0 (1.255.0)	4.0 (3.0/6.0)	2.0 (0.25/3.5)	3.0 (2.25/4.75)	2.0 (3.0/4.75)	3.0 (3.0/4.75)
	Time (s)	12‐month	8.5 (0/24.5)*	4.5 (0/23.0)	10.0 (2.25/25.25)	13.0 (1.75/32.25)	7.5 (0/16.0)	25.0 (12.5/33.0)	33.5 (7.0/55.0)	31.0 (7.0/55.0)	33.5 (5.25/96.5)
		18‐month	13.5 (0.0/44.0)*	10.0 (0.0/44.0)	16.5 (0.0/51.25)	22.5 (14.25/56.5)	33.0 (15.0/55.0)	19.0 (8.75/66.0)	40.0 (23.0/65.0)	34.5 (5.5/61.75)	42.5 (27.0/69.5)
		22‐month	13.5 (0/43.75)*	41.0 (0/74.5)	9.5 (0.75/32.5)*	51.0 (20.0/65.5)	59.0 (39.75/65.5)	29.0 (1.75/63.0)	52.0 (18.0/59.5)	45.5 (16.25/56.0)	53.5 (25.0/64.5)
Object‐location recognition	PI_10 min_	12‐month	0.523 ± 0.025*	0.583 ± 0.041	0.503 ± 0.041*	0.568 ± 0.025	0.601 ± 0.041	0.554 ± 0.041	0.601 ± 0.025	0.634 ± 0.041	0.569 ± 0.041
		18‐month	0.501 ± 0.044*	0.545 ± 0.039*	0.478 ± 0.039*	0.536 ± 0.044*	0.579 ± 0.039	0.523 ± 0.039	0.598 ± 0.044	0.621 ± 0.039	0.556 ± 0.039
		22‐month	0.474 ± 0.038*	0.483 ± 0.040*	0.465 ± 0.040*	0.505 ± 0.038*	0.519 ± 0.040*	0.491 ± 0.040*	0.566 ± 0.038	0.589 ± 0.040	0.544 ± 0.040
	PI_24 hr_	12‐month	0.495 ± 0.029	0.469 ± 0.036	0.541 ± 0.036	0.524 ± 0.029	0.495 ± 0.036	0.573 ± 0.036	0.546 ± 0.029	0.511 ± 0.036	0.583 ± 0.036
		18‐month	0.483 ± 0.037*	0.459 ± 0.042	0.516 ± 0.042	0.528 ± 0.037	0.485 ± 0.042	0.560 ± 0.042	0.553 ± 0.037	0.511 ± 0.042	0.587 ± 0.042
		22‐month	0.446 ± 0.037*	0.424 ± 0.045	0.469 ± 0.045*	0.490 ± 0.037	0.483 ± 0.045	0.508 ± 0.045	0.519 ± 0.037	0.488 ± 0.045	0.549 ± 0.045

Abbreviations: H‐LPS, higher‐dose LPS; L‐LPS, lower‐dose LPS; LPS, lipopolysaccharide.

* Compared to the control group, *p* < .05;

^†^ Compared to the low LPS group, *p* < .05.

#### 3.3.2 Open field

There were significant differences in squares crossed among the LPS groups and the control group only for the combined sexes [*F*_(2, 42)_ = 3.322, *p* = .046]. The H‐LPS mice had less peripheral time than the controls (*p* = .016, Table 1).

#### 3.3.3 Elevated plus maze

There were insignificant differences among the three groups in the time spent on the open arm and number of entries to the open arm for the combined and separated sexes (*p*s > .05, Table 1). The time spent on the open arm of the H‐LPS group was lower than that of the control group (*p* = .040, Table 1).

#### 3.3.4 Object location recognition

During the 10‐min phase, the PI_10 min_ exhibited significant differences among the three groups for the combined sexes [*F*_(2, 42)_ = 4.033, *p* = .025] and females [*F*_(2, 21)_ = 3.897, *p* = .036]. H‐LPS mice had lower PI_10 min_ than the control ones (*p* = .007), which was mainly attributable to the females (*p* = .015, Table 1). During the 24‐hr phase, the LPS treatment effect was not observed for the combined and separated sexes (*p*s > .05, Table 1).

#### 3.3.5 RAWM

##### Learning phase

**Figure 1:**
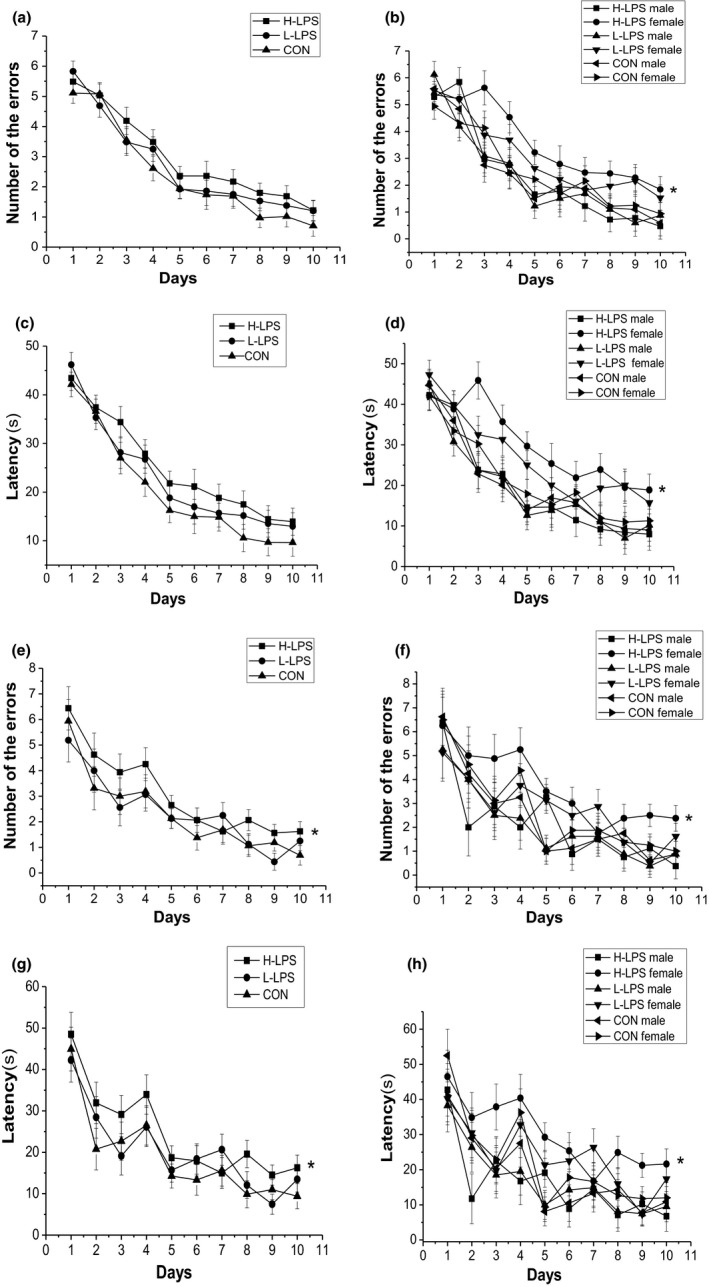
Performance in the radial six‐arm water maze (RAWM) in different groups in the 12‐month CD‐1 mice (*n* = 16 mice/group, eight females and eight males, respectively). Latency (c and d) and number of errors (a and b) during the learning phase; and latency (g and h) and number of errors (e and f) during the memory phase. All values are means ± *SEM*. *p < .05 indicates a significant difference compared to control (CON) mice

The number of errors and latency progressively decreased with days for all mice combined [*F*_(9, 378)_ = 77.370, 71.126; *p*s < .001].There were insignificant differences in the number of errors and latency among the LPS group and the control group for the combined and separated sexes (*p*s > .05). But, the number of errors and latency of H‐LPS female mice were more or longer than that of the control female ones (*p* = .048, .049; see Figure 1a–d).

##### Memory phase

The number of errors and latency progressively decreased over time [*F*_(9, 378)_ = 25.793, 22.848; *p*s < .001]. The LPS treatment effect was not observed for the combined and separated sexes (*p*s > .05). H‐LPS mice had more or longer errors and latency than the control mice (*p* = .047, .041), which was mainly attributable to the females (*p* = .042, .046; see Figure 1e–h). The sex and interactions of group × sex, group × day, sex × day, and group × sex × day had insignificant effects in these trials (*p*s > .05).

### 3.4 Behaviors in the 18‐month‐old mice

#### 3.4.1 Nesting task

The LPS treatment affected the score of nesting (*χ* ^2^ = 7.379, *p* = .025). H‐LPS mice had a lower nesting score than the control mice (*χ* ^2^ = 5.822, *p* = .016), which was contributable to the males (*χ* ^2^ = 4.204, *p* = .040) and the females (*χ* ^2^ = 4.527, *p* = .033, Table 1).

#### 3.4.2 Beam walking task

The LPS treatment affected the balance time (*χ* ^2^ = 9.549, *p* = .023). The balance time of H‐LPS mice was significantly shorter than that of controls for the combined sexes (*χ* ^2^ = 10.295, *p* = .011) and the males (*χ* ^2^ = 7.873, *p* = .005, Table 1).

#### 3.4.3 Open field

The LPS treatment affected the peripheral time and squares crossed [*F*_(2, 42)_ = 6.626, 3.950; *p*s < .05]. H‐LPS mice showed a longer peripheral time than the controls (*p* = .029) and L‐LPS mice (*p* = .024), and less squares crossed than the controls (*p* = .008). H‐LPS female mice had a longer peripheral time than the control female mice (*p* = .005). H‐LPS male mice had a longer peripheral time than the controls (*p* = .004) and L‐LPS mice (*p* = .005). H‐LPS male mice showed less squares crossed than the controls (*p* = .017, Table 1).

#### 3.4.4 Elevated plus maze

The LPS treatment did not affect the time spent on the open arm and number of entries to the open arm for the combined sexes (*χ* ^2^ = 5.710, 3.632; *p*s < .05). But, H‐LPS mice showed shorter time spent on the open arm than the controls (*p* = .024, Table 1).

#### 3.4.5 Object location recognition

During the 10‐min phase, the LPS treatment affected PI_10 min_ [*F*_(2, 42)_ = 7.014, *p* < .001]. Both H‐LPS and L‐LPS mice showed a lower PI_10 min_ than the controls (*p* = .003, .032). H‐LPS female and male mice had a lower PI_10 min_ than the same‐sex control mice (*p* = .004, .019). During the 24‐hr phase, the LPS treatment also affected PI_24 hr_ [*F*_(2, 42)_ = 3.470, *p* = .040]. Only H‐LPS mice exhibited lower PI_24 hr_ than the control mice (*p* = .015, Table 1).

#### 3.4.6 Radial six‐arm water maze

##### Learning phase

**Figure 2:**
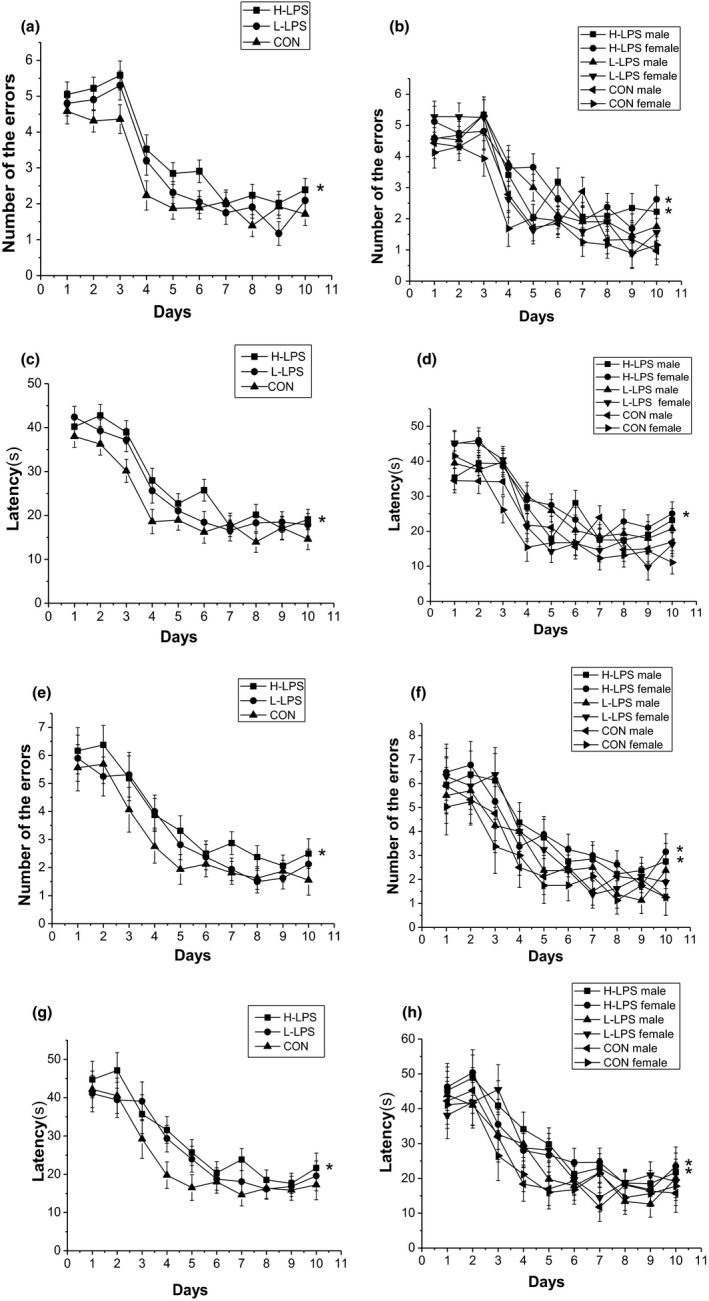
Performance in the radial six‐arm water maze (RAWM) in different groups in the 18‐month CD‐1 mice (*n* = 16 mice/group, eight females and eight males, respectively). Latency (c and d) and number of errors (a and b) during the learning phase; and latency (g and h) and number of errors (e and f) during the memory phase. All values are means ± *SEM*. *p < .05 indicates a significant difference compared to control (CON) mice

The number of errors and latency progressively decreased daily for all mice combined [*F*_(9, 378)_ = 53.736, 53.693; *p*s < .001]. There were significant differences in the number of errors and latency among LPS and the control groups for the combined sexes [*F*_(2, 42)_ = 4.972, 3.754; *p* = .012, .032]. More errors and longer latency in H‐LPS mice than that of the controls (*p* = .03, .010). The LPS effect of the number of errors was contributable to the females (*p* = .008) and males (*p* = .047), but the LPS effect of the latency was only contributable to the females (*p* = .004), and marginally to the males (*p* = .063; see Figure 2a–d).

##### Memory phase

The number of errors and latency progressively decreased over time [*F*_(9, 378)_ = 23.943, 25.054; *p*s < .001]. The LPS treatment effect was observed in the number of errors [*F*_(2, 42)_ = 4.146, *p* = .024] and marginally in the latency [*F*_(2, 42)_ = 3.108, *p* = .087] for the combined sexes. H‐LPS mice had more errors and longer latency than the control mice (*p* = .015, .041), which was attributable to the females and females (*p*s < .05; see Figure 2e–h). The sex and interactions of group × sex, group × day, sex × day, and group × sex × day had no significant effects in these trials (*p*s > .05).

### 3.5 Behaviors in the 22‐month‐old mice

#### 3.5.1 Nesting

The performance in the nesting task is presented in Table 1. There were significant differences among the three groups for the combined sexes (*χ* ^2^ = 7.734, *p* = .023). Both H‐LPS mice (*χ* ^2^ = 7.362, *p* = .007) and L‐PLS mice (*χ* ^2^ = 5.417, *p* = .036) had lower scores than the control ones. Both the H‐LPS males (*χ* ^2^ = 4.894, *p* = .027) and L‐LPS males (*χ* ^2^ = 5.126, *p* = .047) showed lower scores than the same‐sex control mice, and only H‐LPS females had lower scores than the control females (*χ* ^2^ = 5.902, *p* = .015).

#### 3.5.2 Beam walking

There were significant differences in the balance time among the LPS mice and the control mice for the combined sexes (*χ* ^2^ = 10.066, *p* = .003). Both H‐LPS mice and L‐PLS mice had lower balance time than the control mice (*χ* ^2^ = 12.060, 9.463; *p*s < .05). Meanwhile, both the H‐LPS males and L‐LPS males showed lower scores than the same‐sex control mice (*χ* ^2^ = 5.426, 6.113; *p* = .020, .048), and L‐LPS females had lower scores than the control females (*χ* ^2^ = 7.50, *p* = .006; see Table 1).

#### 3.5.3 Open field

There were significant differences in the peripheral time and squares crossed among the LPS groups and the control group for the combined sexes [*F*_(2, 42)_ = 3.897, 3.557; *p*s < .05]. H‐LPS exhibited a longer peripheral time and less squares crossed than the control ones (*p*s < .05), which were, respectively, attributable to the females (*p* = .011) and males (*p* = .038; see Table 1).

#### 3.5.4 Elevated plus maze

There were insignificant differences among the three groups in the time spent on the open arm and number of entries to the open arm for the combined sexes (*χ* ^2^ = 4.777, 5.269; *p* = .092, .072). However, H‐LPS mice had less time spent on the open arm and number of entries to the open arm than the control mice (*χ* ^2^ = 4.801, 5.107; *p* = .028, .024), which was mainly attributable to the females (*p*s < .05; see Table 1).

#### 3.5.5 Object location recognition

During the 10‐min phase, there were significant differences among the LPS‐treated mice and the control mice [*F*_(2, 42)_ = 9.660, *p*s < .001]. Both H‐LPS and L‐LPS mice had a lower PI_10 min_ than the controls (*p* = .001, .003), which was attributable to the females and males (*p*s < .05). During the 24‐hr phase, the LPS treatment effect was observed from the combined sexes [*F*_(2, 42)_ = 5.180, *p* = .010], and females [*F*_(2, 21)_ = 3.644, *p* = .044]. H‐LPS mice had a lower PI_24 hr_ than the control mice (*p* = .003), which was contributable to the females (*p* = .015; see Table 1).

#### 3.5.6 Radial six‐arm water maze

##### Learning phase

**Figure 3:**
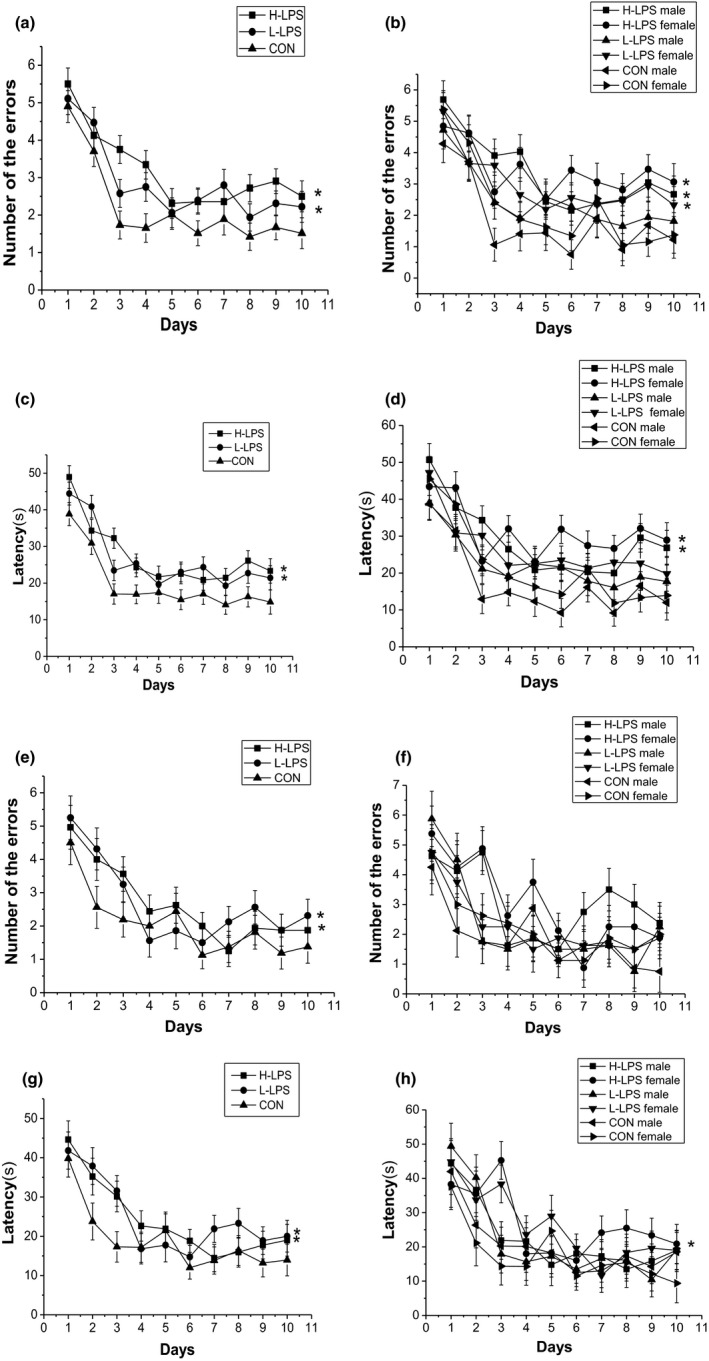
Performance in the radial six‐arm water maze (RAWM) in different groups in the 22‐month CD‐1 mice (*n* = 16 mice/group, eight females and eight males, respectively). Latency (c and d) and number of errors (a and b) during the learning phase; and latency (g and h) and number of errors (e and f) during the memory phase. All values are means ± *SEM*. *p < .05 indicates a significant difference compared to control (CON) mice

The number of errors and latency progressively decreased every day for all mice combined [*F*_(9, 378)_ = 25.882, 34.062; *p*s < .001]. There were significant differences in the number of errors and latency among the three groups for the combined sexes [*F*_(2, 42)_ = 6.531, 6.438; *p* = .003, .004] and the females [*F*_(2, 21)_ = 5.946, 5.776; *p* = .010, .010], but not for the males [*F*_(2, 21)_ = 3.109, 2.817; *p* = .084, .105]. There were more errors and longer latency in H‐LPS mice than that of the control mice for the combined and separated sexes (*p*s < .05). Additionally, there were more errors and longer latency in L‐LPS mice than that of the controls only for the combined sexes and the females (*p*s < .05; see Figure 3a–d).

##### Memory phase

The number of errors and latency progressively decreased over time for all the mice [*F*_(9, 378)_ = 13.728, 15.551; *p*s < .001]. The LPS treatment effects were significant in the number of errors and latency for the combined sexes [*F*_(2, 42)_ = 6.871, 4.618; *p* = .012, .015], but not for the females [*F*_(2, 21)_ = 2.172, 2.621; *p* = .139, .096,] and the males [*F*_(2, 21)_ = 0.995, 2.229; *p* = .386, .132]. H‐LPS group had significantly more errors and longer latency than the CON group for the combined sexes (*p*s < .05), and longer latency for the females (*p* = .039). Similarly, L‐LPS mice showed more errors and longer latency than the CON mice for the combined sexes (*p*s < .05). The sex and interactions of group × sex, group × day, sex × day, and group × sex × day had insignificant effects in these trials (*p*s > .05; see Figure 3e–h).

### 3.6 The results of histopathological staining

**Figure 4:**
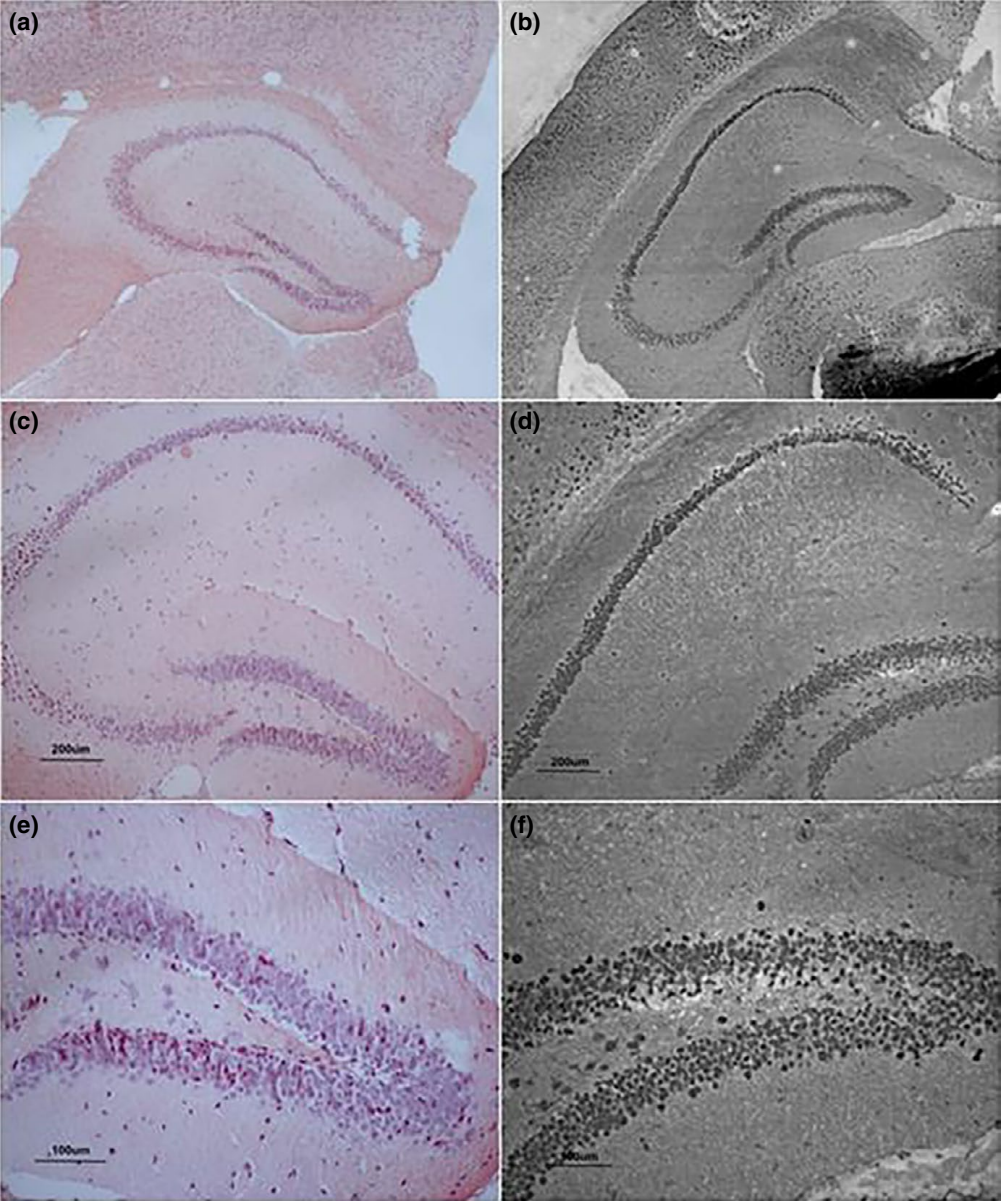
Congo red and Bielschowsky staining. (a, c, and e) stained by Congo red; and (b, d, and f) stained by Bielschowsky in 22‐month CD‐1 mice; (a and b) represent the higher‐dose lipopolysaccharide (H‐LPS) group, (c and d) represent the lower‐dose lipopolysaccharide (L‐LPS) group, and (e and f) represent the control (CON) group. (a and b) under 40× magnification, (c and d) under 100× magnification, (e and f) under 200× magnification

In the Congo red staining, cell nuclei and the background were, respectively, bluish violet and light red. Amyloid plaque was not observed in the hippocampus of the older control or LPS‐treated mice. In the Bielschowsky staining, cell nuclei and nerve fibers displayed as deep black, and the background was light black. Sections obtained from LPS‐treated mice, and older mice showed no neurofibrillary tangles (see Figure 4).

### 3.7 Levels of Aβ_42_, p‐tau, and GFAP in different hippocampal layers

In this study, Aβ_42_, p‐tau, and GFAP were shown in each layer in the dorsal hippocampus (Tables 2, 3, 4, 5). The Aβ_42_ and p‐tau expressions were observed in every layer of the hippocampus. The GFAP‐positive astrocytes were morphologically enlarged in the older hippocampus. Because there were insignificant LPS treatment effects on the levels of Aβ_42_, p‐tau, and GFAP in the 1‐ and 6‐month‐old mice (*p*s > .05), the results are not described further here.

**Table 2:** The relative levels of Aβ and p‐tau in different hippocampal sublayer in the 12‐month CD‐1 mice

Proteins		Regions	H‐LPS mice			L‐LPS mice			CON mice		
			All mice	males	females	All mice	males	females	All mice	males	females
Aβ	DG	GL	0.050 ± 0.002	0.049 ± 0.003	0.051 ± 0.003	0.050 ± 0.003	0.052 ± 0.004	0.049 ± 0.004	0.047 ± 0.002	0.049 ± 0.003	0.046 ± 0.004
		HL	0.034 ± 0.002*	0.035 ± 0.003	0.033 ± 0.003*	0.031 ± 0.003	0.033 ± 0.005	0.028 ± 0.005	0.024 ± 0.003	0.024 ± 0.003	0.023 ± 0.004
		MS	0.042 ± 0.002*	0.040 ± 0.003	0.044 ± 0.002*	0.038 ± 0.003	0.036 ± 0.004	0.039 ± 0.004	0.036 ± 0.002	0.039 ± 0.003	0.032 ± 0.003
	CA1	MS	0.028 ± 0.002	0.029 ± 0.003	0.026 ± 0.003	0.028 ± 0.003	0.030 ± 0.005	0.026 ± 0.005	0.027 ± 0.003	0.023 ± 0.003	0.032 ± 0.004
		RS	0.042 ± 0.001*	0.043 ± 0.002	0.041 ± 0.001	0.039 ± 0.001	0.038 ± 0.002	0.041 ± 0.002	0.038 ± 0.001	0.039 ± 0.002	0.037 ± 0.002
		PL	0.078 ± 0.003	0.084 ± 0.004	0.073 ± 0.003	0.078 ± 0.004	0.071 ± 0.005	0.080 ± 0.005	0.077 ± 0.003	0.079 ± 0.004	0.075 ± 0.004
		OS	0.033 (0.028, 0.035)	0.033 (0.028, 0.035)	0.030 (0.024, 0.035)	0.032 (0.030, 0.038)	0.032 (0.031, 0.034)	0.034 (0.029, 0.039)	0.031 (0.030, 0.035)	0.031 (0.030, 0.034)	0.033 (0.024, 0.037)
	CA3	MS	0.038 (0.024, 0.040)	0.038 (0.038, 0.046)	0.025 (0.019, 0.040)	0.034 (0.030, 0.039)	0.034 (0.033, 0.036)	0.035 (0.029, 0.040)	0.035 (0.030, 0.038)	0.031 (0.029, 0.034)	0.038 (0.035, 0.045)
		LS	0.042 ± 0.002*	0.044 ± 0.003	0.041 ± 0.002	0.038 ± 0.003	0.037 ± 0.004	0.039 ± 0.004	0.035 ± 0.002	0.035 ± 0.003	0.035 ± 0.003
		PL	0.057 ± 0.003	0.058 ± 0.004	0.057 ± 0.004	0.062 ± 0.004	0.067 ± 0.006	0.057 ± 0.006	0.057 ± 0.003	0.056 ± 0.004	0.057 ± 0.005
		OS	0.037 ± 0.003	0.045 ± 0.005	0.028 ± 0.004	0.031 ± 0.005	0.026 ± 0.007	0.036 ± 0.007	0.030 ± 0.004	0.034 ± 0.005	0.025 ± 0.005
p‐tau	DG	GL	0.066 ± 0.003	0.066 ± 0.004	0.066 ± 0.004	0.066 ± 0.003	0.065 ± 0.004	0.067 ± 0.004	0.064 ± 0.002	0.063 ± 0.004	0.064 ± 0.004
		HL	0.037 ± 0.002	0.038 ± 0.003	0.036 ± 0.003	0.035 ± 0.002	0.035 ± 0.003	0.035 ± 0.003	0.035 ± 0.002	0.037 ± 0.003	0.033 ± 0.003
		MS	0.041 ± 0.002*	0.040 ± 0.002	0.042 ± 0.002	0.037 ± 0.002	0.036 ± 0.002	0.039 ± 0.002	0.036 ± 0.002	0.035 ± 0.002	0.036 ± 0.002
	CA1	MS	0.032 ± 0.002	0.033 ± 0.003	0.030 ± 0.003	0.028 ± 0.002	0.030 ± 0.003	0.027 ± 0.003	0.027 ± 0.002	0.027 ± 0.003	0.027 ± 0.003
		RS	0.040 ± 0.001*	0.042 ± 0.002	0.039 ± 0.002	0.037 ± 0.001	0.037 ± 0.002	0.037 ± 0.002	0.036 ± 0.001	0.036 ± 0.002	0.035 ± 0.002
		PL	0.094 ± 0.003*	0.095 ± 0.005	0.093 ± 0.005	0.092 ± 0.004	0.093 ± 0.005	0.091 ± 0.005	0.083 ± 0.003	0.083 ± 0.005	0.082 ± 0.005
		OS	0.029 (0.028, 0.034)	0.032 (0.023, 0.035)	0.028 (0.027, 0.030)	0.028 (0.024, 0.032)	0.030 (0.026, 0.035)	0.034 (0.029, 0.039)	0.029 (0.020, 0.035)	0.029 (0.020, 0.035)	0.030 (0.021, 0.035)

Abbreviations: CON, control; H‐LPS, higher‐dose LPS; L‐LPS, lower‐dose LPS; LPS, lipopolysaccharide.

* Compared to the control group, *p* < .05.

**Table 3:** The relative levels of Aβ and tau in different hippocampal sublayer in the 18‐month CD‐1 mice

Proteins		Regions	H‐LPS mice			L‐LPS mice			CON mice		
			All mice	Males	Females	All mice	Males	Females	All mice	Males	Females
Aβ	DG	GL	0.057 ± 0.005	0.061 ± 0.007	0.053 ± 0.007	0.051 ± 0.003	0.049 ± 0.004	0.053 ± 0.005	0.050 ± 0.003	0.050 ± 0.004	0.050 ± 0.005
		HL	0.036 ± 0.002*	0.032 ± 0.003	0.038 ± 0.003*	0.029 ± 0.002	0.027 ± 0.002	0.031 ± 0.002	0.026 ± 0.002	0.027 ± 0.002	0.025 ± 0.002
		MS	0.043 ± 0.002*	0.043 ± 0.003*	0.043 ± 0.003*	0.040 ± 0.001*	0.038 ± 0.002	0.043 ± 0.002*	0.035 ± 0.002	0.035 ± 0.002	0.035 ± 0.002
	CA1	MS	0.032 ± 0.002*	0.033 ± 0.003	0.031 ± 0.003	0.028 ± 0.001	0.028 ± 0.001	0.028 ± 0.001	0.027 ± 0.001	0.026 ± 0.001	0.027 ± 0.002
		RS	0.044 ± 0.001*	0.044 ± 0.002	0.044 ± 0.002*	0.039 ± 0.001	0.040 ± 0.001	0.038 ± 0.001	0.038 ± 0.001	0.038 ± 0.001	0.037 ± 0.001
		PL	0.085 (0.077, 0.095)*	0.084 (0.077, 0.091)	0.088 (0.079, 0.097)*	0.079 (0.073, 0.086)	0.082 (0.068, 0.091)	0.077 (0.074, 0.079)	0.076 (0.071, 0.082)	0.079 (0.072, 0.082)	0.076 (0.072, 0.081)
		OS	0.035 ± 0.002	0.037 ± 0.003	0.034 ± 0.003	0.035 ± 0.001	0.034 ± 0.002	0.036 ± 0.002	0.034 ± 0.001	0.036 ± 0.002	0.032 ± 0.002
	CA3	MS	0.039 ± 0.002*	0.039 ± 0.002*	0.038 ± 0.002*	0.036 ± 0.001	0.034 ± 0.002	0.039 ± 0.001*	0.035 ± 0.001	0.036 ± 0.002	0.034 ± 0.001
		LS	0.045 ± 0.002* ^,^ †	0.043 ± 0.003	0.048 ± 0.003* ^,^ †	0.039 ± 0.003	0.041 ± 0.002	0.037 ± 0.002	0.036 ± 0.002	0.036 ± 0.002	0.037 ± 0.002
		PL	0.070 ± 0.004*	0.072 ± 0.006	0.069 ± 0.006*	0.065 ± 0.003	0.062 ± 0.003	0.069 ± 0.004*	0.058 ± 0.003	0.060 ± 0.003	0.055 ± 0.004
		OS	0.041 ± 0.002* ^,^ †	0.040 ± 0.003*	0.042 ± 0.003* ^,^ †	0.034 ± 0.002	0.034 ± 0.002	0.034 ± 0.002	0.032 ± 0.001	0.033 ± 0.002	0.032 ± 0.002
p‐tau	DG	GL	0.074 ± 0.002*	0.074 ± 0.002	0.074 ± 0.002*	0.070 ± 0.002	0.070 ± 0.002	0.069 ± 0.002	0.069 ± 0.002	0.071 ± 0.002	0.067 ± 0.002
		HL	0.041 ± 0.001*	0.041 ± 0.002	0.040 ± 0.002	0.037 ± 0.001	0.038 ± 0.002	0.037 ± 0.002	0.037 ± 0.001	0.037 ± 0.002	0.036 ± 0.002
		MS	0.043 ± 0.001* ^,^ †	0.044 ± 0.001* ^,^ †	0.042 ± 0.001*	0.039 ± 0.001	0.039 ± 0.001	0.040 ± 0.001	0.038 ± 0.001	0.037 ± 0.001	0.038 ± 0.001
	CA1	MS	0.036 ± 0.002* ^,^ †	0.038 ± 0.003*	0.035 ± 0.003	0.031 ± 0.002	0.032 ± 0.003	0.030 ± 0.003	0.028 ± 0.002	0.029 ± 0.003	0.027 ± 0.003
		RS	0.044 ± 0.001*	0.045 ± 0.002*	0.043 ± 0.002	0.043 ± 0.001*	0.044 ± 0.002*	0.042 ± 0.002	0.039 ± 0.001	0.038 ± 0.002	0.040 ± 0.002
		PL	0.100 ± 0.004*	0.099 ± 0.006*	0.100 ± 0.006	0.093 ± 0.004	0.095 ± 0.006	0.091 ± 0.006	0.086 ± 0.004	0.085 ± 0.006	0.087 ± 0.006
		OS	0.032 ± 0.002*	0.030 ± 0.002	0.033 ± 0.002*	0.030 ± 0.002*	0.030 ± 0.002	0.029 ± 0.002	0.024 ± 0.002	0.024 ± 0.002	0.025 ± 0.002
	CA3	MS	0.046 ± 0.003	0.047 ± 0.004	0.045 ± 0.004	0.042 ± 0.003	0.040 ± 0.004	0.043 ± 0.004	0.039 ± 0.003	0.039 ± 0.004	0.040 ± 0.004
		LS	0.049 ± 0.002*	0.048 ± 0.003	0.049 ± 0.003	0.043 ± 0.002	0.043 ± 0.003	0.044 ± 0.003	0.042 ± 0.002	0.043 ± 0.003	0.041 ± 0.003
		PL	0.078 (0.073, 0.090)*	0.078 (0.075, 0.082)	0.082 (0.072, 0.097)*	0.076 (0.067, 0.081)	0.076 (0.067, 0.080)	0.077 (0.067, 0.085)	0.067 (0.061, 0.081)	0.073 (0.053, 0.086)	0.068 (0.061, 0.077)
		OS	0.035 (0.026, 0.038)	0.037 (0.027, 0.038)	0.034 (0.021, 0.036)	0.033 (0.022, 0.036)	0.034 (0.025, 0.036)	0.030 (0.024, 0.035)	0.030 (0.022, 0.032)	0.027 (0.021, 0.035)	0.032 (0.027, 0.034)

Abbreviations: CON, control; H‐LPS, higher‐dose LPS; L‐LPS, lower‐dose LPS; LPS, lipopolysaccharide.

* Compared to the control group, *p* < .05;

^†^ Compared to the low LPS group, *p* < .05.

**Table 4:** The relative levels of Aβ and tau in different hippocampal sublayer in the 22‐month CD‐1 mice

Proteins		Regions	H‐LPS mice			L‐LPS mice			CON mice		
			All mice	Males	Females	All mice	Males	Females	All mice	Males	Females
Aβ	DG	GL	0.058 ± 0.001* ^,^ †	0.059 ± 0.002*	0.058 ± 0.002†	0.053 ± 0.001	0.056 ± 0.002	0.051 ± 0.002	0.052 ± 0.001	0.050 ± 0.002	0.054 ± 0.002
		HL	0.036 ± 0.001* ^,^ †	0.035 ± 0.002*	0.036 ± 0.002* ^,^ †	0.032 ± 0.001*	0.033 ± 0.002	0.031 ± 0.002	0.028 ± 0.001	0.028 ± 0.002	0.028 ± 0.002
		MS	0.045 ± 0.002*	0.046 ± 0.002*	0.045 ± 0.002*	0.042 ± 0.002*	0.042 ± 0.003	0.041 ± 0.003	0.036 ± 0.002	0.035 ± 0.003	0.036 ± 0.003
	CA1	MS	0.038 ± 0.002*	0.037 ± 0.003	0.038 ± 0.003	0.032 ± 0.002	0.032 ± 0.003	0.032 ± 0.003	0.031 ± 0.002	0.030 ± 0.003	0.032 ± 0.003
		RS	0.043 ± 0.002*	0.043 ± 0.002	0.043 ± 0.002	0.042 ± 0.002*	0.041 ± 0.002	0.043 ± 0.003	0.037 ± 0.002	0.036 ± 0.003	0.037 ± 0.002
		PL	0.088 ± 0.003*	0.087 ± 0.004*	0.088 ± 0.003	0.082 ± 0.003	0.080 ± 0.004	0.083 ± 0.004	0.079 ± 0.003	0.078 ± 0.004	0.080 ± 0.004
		OS	0.038 ± 0.002	0.038 ± 0.003	0.038 ± 0.003	0.035 ± 0.002	0.037 ± 0.003	0.034 ± 0.003	0.035 ± 0.002	0.034 ± 0.003	0.036 ± 0.003
	CA3	MS	0.043 ± 0.002*	0.041 ± 0.003	0.044 ± 0.003*	0.040 ± 0.002	0.040 ± 0.003	0.039 ± 0.003	0.036 ± 0.002	0.036 ± 0.003	0.036 ± 0.003
		LS	0.049 ± 0.002*	0.050 ± 0.002*	0.048 ± 0.002*	0.044 ± 0.002*	0.044 ± 0.003	0.043 ± 0.003	0.037 ± 0.002	0.038 ± 0.003	0.037 ± 0.003
		PL	0.081 ± 0.003* ^,^ †	0.082 ± 0.004* ^,^ †	0.081 ± 0.004* ^,^ †	0.066 ± 0.003	0.066 ± 0.004	0.066 ± 0.004	0.061 ± 0.003	0.062 ± 0.004	0.059 ± 0.004
		OS	0.042 ± 0.001*	0.042 ± 0.002*	0.041 ± 0.002	0.039 ± 0.002*	0.039 ± 0.002	0.039 ± 0.002	0.035 ± 0.002	0.034 ± 0.002	0.036 ± 0.002
p‐tau	DG	GL	0.080 ± 0.002*	0.080 ± 0.003	0.079 ± 0.003	0.076 ± 0.002	0.077 ± 0.003	0.074 ± 0.003	0.072 ± 0.002	0.073 ± 0.003	0.072 ± 0.003
		HL	0.046 ± 0.002*	0.047 ± 0.003	0.045 ± 0.003	0.042 ± 0.002	0.043 ± 0.003	0.041 ± 0.003	0.039 ± 0.002	0.040 ± 0.003	0.039 ± 0.003
		MS	0.050 ± 0.002*	0.051 ± 0.003*	0.050 ± 0.003*	0.047 ± 0.002*	0.047 ± 0.003*	0.047 ± 0.003	0.041 ± 0.002	0.041 ± 0.003	0.041 ± 0.003
	CA1	MS	0.039 ± 0.002*	0.038 ± 0.003	0.040 ± 0.003* ^,^ †	0.034 ± 0.002	0.036 ± 0.003	0.033 ± 0.003	0.031 ± 0.002	0.032 ± 0.003	0.031 ± 0.003
		RS	0.048 ± 0.002*	0.047 ± 0.003	0.049 ± 0.003	0.047 ± 0.002*	0.048 ± 0.003	0.047 ± 0.003	0.041 ± 0.002	0.040 ± 0.003	0.042 ± 0.003
		PL	0.109 ± 0.004* ^,^ †	0.112 ± 0.005*	0.105 ± 0.005*	0.096 ± 0.004	0.097 ± 0.005	0.094 ± 0.005	0.089 ± 0.004	0.088 ± 0.005	0.090 ± 0.005
		OS	0.036 ± 0.001*	0.037 ± 0.002*	0.035 ± 0.002*	0.034 ± 0.001*	0.034 ± 0.002*	0.034 ± 0.002*	0.028 ± 0.001	0.028 ± 0.002	0.027 ± 0.002
	CA3	MS	0.050 ± 0.002*	0.052 ± 0.003*	0.047 ± 0.003	0.044 ± 0.002	0.043 ± 0.003	0.046 ± 0.003	0.041 ± 0.002	0.041 ± 0.003	0.041 ± 0.003
		LS	0.053 ± 0.002* ^,^ †	0.055 ± 0.003* ^,^ †	0.051 ± 0.003*	0.046 ± 0.002	0.046 ± 0.003	0.046 ± 0.003	0.043 ± 0.002	0.045 ± 0.003	0.041 ± 0.003
		PL	0.084 ± 0.003*	0.081 ± 0.005*	0.086 ± 0.005	0.077 ± 0.003	0.076 ± 0.005	0.079 ± 0.005	0.072 ± 0.003	0.071 ± 0.005	0.072 ± 0.005
		OS	0.029 ± 0.002	0.031 ± 0.003	0.027 ± 0.003	0.029 ± 0.002	0.031 ± 0.003	0.027 ± 0.003	0.026 ± 0.002	0.027 ± 0.003	0.026 ± 0.003

Abbreviations: CON, control; H‐LPS, higher‐dose LPS; L‐LPS, lower‐dose LPS; LPS, lipopolysaccharide.

* Compared to the control group, *p* < .05;

^†^ Compared to the low LPS group, *p* < .05.

**Table 5:** The relative levels of GFAP in different hippocampal subregion in the middle‐aged and old CD‐1 mice

Region	H‐LPS mice			L‐LPS mice			CON mice		
	All mice	Males	Females	All mice	Males	Females	All mice	Males	Females
12 months									
DG	0.037 ± 0.007	0.033 ± 0.009	0.040 ± 0.009	0.034 ± 0.007	0.031 ± 0.009	0.036 ± 0.009	0.031 ± 0.007	0.028 ± 0.009	0.033 ± 0.009
CA1	0.029 ± 0.005	0.029 ± 0.007	0.028 ± 0.007	0.025 ± 0.005	0.022 ± 0.007	0.029 ± 0.007	0.028 ± 0.005	0.024 ± 0.007	0.031 ± 0.007
CA3	0.051 ± 0.004* ^,^ †	0.052 ± 0.006* ^,^ †	0.049 ± 0.006*	0.038 ± 0.004	0.038 ± 0.006	0.037 ± 0.006	0.037 ± 0.004	0.036 ± 0.006	0.038 ± 0.006
18 months									
DG	0.068 ± 0.009	0.065 ± 0.010	0.070 ± 0.010	0.059 ± 0.009	0.055 ± 0.010	0.067 ± 0.010	0.054 ± 0.009	0.053 ± 0.010	0.057 ± 0.010
CA1	0.061 ± 0.006*	0.067 ± 0.007*	0.058 ± 0.007	0.047 ± 0.006	0.052 ± 0.007	0.043 ± 0.007	0.034 ± 0.006	0.031 ± 0.007	0.036 ± 0.007
CA3	0.077 ± 0.008*	0.071 ± 0.009*	0.083 ± 0.009*	0.063 ± 0.008	0.057 ± 0.009	0.065 ± 0.009	0.055 ± 0.008	0.051 ± 0.009	0.059 ± 0.009
22 months									
DG	0.094 ± 0.008*	0.098 ± 0.009*	0.087 ± 0.009	0.089 ± 0.008	0.085 ± 0.009	0.094 ± 0.09	0.069 ± 0.008	0.064 ± 0.009	0.072 ± 0.009
CA1	0.087 ± 0.006* ^,^ †	0.084 ± 0.007* ^,^ †	0.096 ± 0.007* ^,^ †	0.062 ± 0.006	0.056 ± 0.007	0.065 ± 0.007	0.055 ± 0.006	0.051 ± 0.007	0.060 ± 0.007
CA3	0.091 ± 0.006*	0.097 ± 0.008*	0.088 ± 0.008*	0.083 ± 0.006*	0.081 ± 0.008*	0.085 ± 0.008	0.062 ± 0.006	0.056 ± 0.008	0.065 ± 0.008

Abbreviations: CON, control; H‐LPS, higher‐dose LPS; L‐LPS, lower‐dose LPS; LPS, lipopolysaccharide.

* Compared to the control group, *p* < .05;

^†^ Compared to the low LPS group, *p* < .05.

At the age of 12 months, LPS treatment effects on the levels of Aβ_42_ and p‐tau were significant in different hippocampal layers; that is, Aβ_42_ in DG‐HL and CA3‐LS [*F*_(2, 42)_ = 4.295, 4.147; *p* = .035, .039] and p‐tau in CA1‐RS and CA1‐PL [*F*_(2, 42)_ = 3.919, 3.614; *p* = .039, .048; Table 2], and marginally significant on the level of GFAP in CA3 [*F*_(2, 42)_ = 3.244, *p* = .063; Table 5]. Compared to the same‐age CON, H‐LPS had significantly elevated Aβ_42_ levels in DG‐HL, DG‐MS, CA1‐RS, and CA3‐LS (*p*s < .05; Table 2); p‐tau levels in DG‐MS, CA1‐RS, and CA1‐PL (*p*s < .05; Table 2); and GFAP level in CA3 (*p*s < .05; Table 5). In addition, H‐LPS mice also had higher levels of GFAP than L‐LPS ones in CA3 (*p* = .026; Table 5). Only the H‐LPS female mice had higher levels of Aβ_42_ in DG‐HL and DG‐MS than the same‐sex and same‐age CON ones [*F*_(2, 21)_ = 5.159, 5.853; *p* = .042, .030; Table 2]. Both the males and females contributed to the LPS treatment effects of GFAP levels in CA3 (*p*s < .05; Table 5).

For the 18‐month‐old mice, the significantly increased level of Aβ_42_, p‐tau, and GFAP occurred in most hippocampal layers; that is, Aβ_42_ levels in DG‐HL, DG‐MS, CA1‐RS, CA3‐LS, CA3‐PL, and CA3‐OS (*p*s < .05, Table 3); p‐tau levels in DG‐MS, CA1‐MS, CA1‐RS, and CA1‐OS (*p*s < .05, Table 3); and GFAP levels in CA1 and CA3 [*F*_(2, 42)_ = 4.480, 4.070; *p* = .030, .035; Table 5]. H‐LPS mice held higher levels of Aβ_42_ in all the aforementioned subregion layers and CA1‐PL (*p*s < .05) than the CON mice, and higher levels in the CA3‐LS, CA3‐OS than L‐LPS ones (*p*s < .05). The L‐LPS group had higher levels of Aβ_42_ in the DG‐MS than the CON group (*p* = .025). Compared to the same‐sex CON, the H‐LPS males had significantly increased Aβ_42_ in DG‐MS, CA3‐MS, and CA3‐OS (*p*s < .05), and so did the H‐LPS females in almost hippocampal layers except for DG‐GL, CA1‐MS, and CA1‐OS. In addition, the H‐LPS females showed higher levels of Aβ_42_ than L‐LPS females in CA3‐LS and CA3‐OS (*p*s < .05). Relative to the same‐age CON, H‐LPS mice had significantly increased p‐tau in every subregion layers in DG, CA1, and CA3‐(LS and PL; *p*s < .05). L‐LPS mice had higher levels of p‐tau than CON ones in CA1‐RS and CA1‐OS and lower levels of p‐tau than H‐LPS mice in DG‐MS and CA1‐MS (*p*s < .05). The H‐LPS male mice had significantly higher levels of p‐tau in DG‐MS, CA1‐MS, CA1‐RS, and CA1‐RL than CON male ones (*p*s < .05) and higher levels of p‐tau in DG‐MS than the L‐LPS male mice (*p* = .036). In addition, H‐LPS mice showed higher levels of p‐tau in DG‐GL, DG‐MS, CA1‐OS, and CA3‐PL than CON female mice (*p*s < .05). H‐LPS mice had higher levels of GFAP than CON ones in CA1 and CA3 (*p* = .008, .015), which were contributable to the males and females (*p*s < .05).

**Figure 5:**
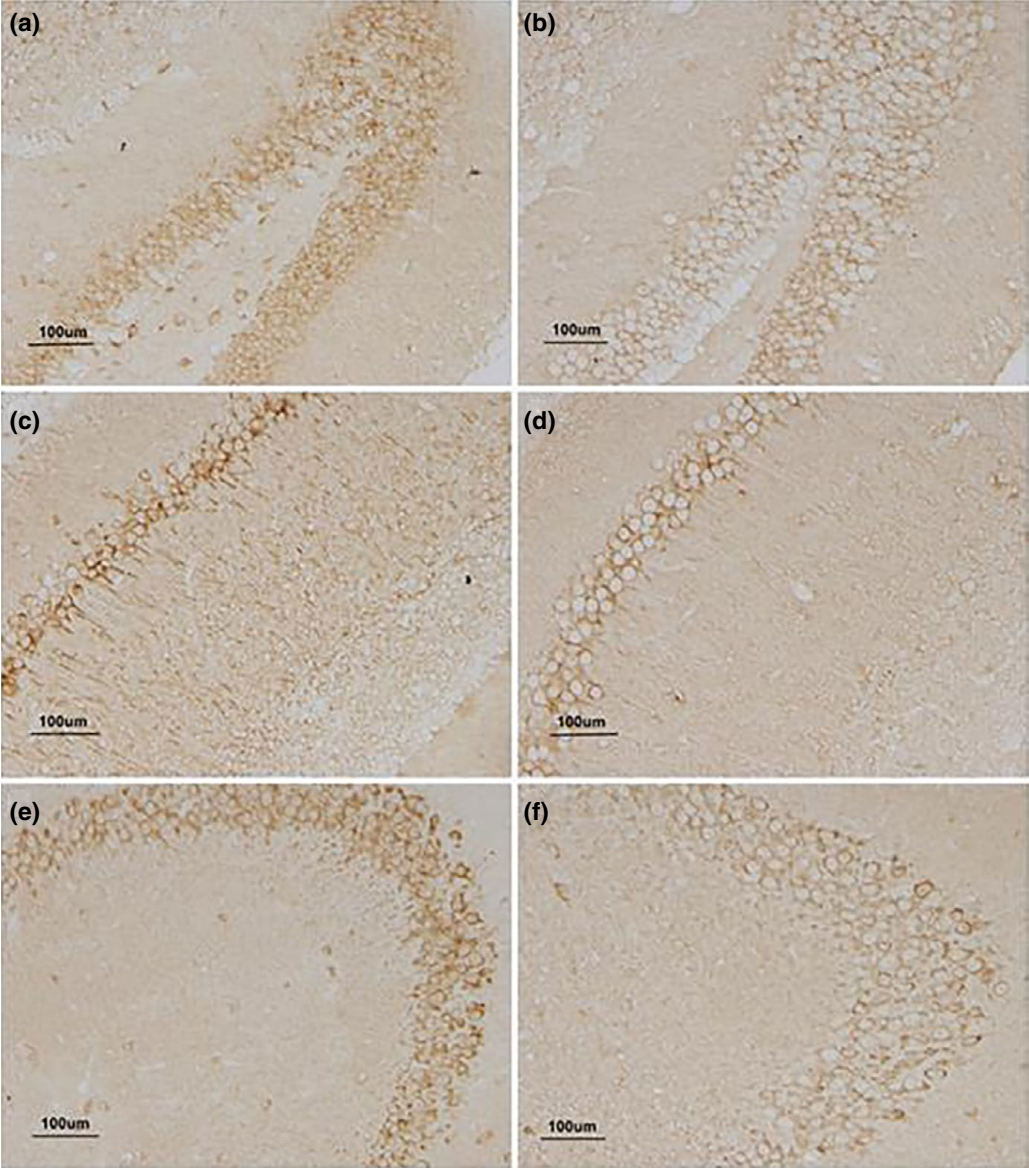
The expression of Aβ in the hippocampus of 22‐month CD‐1 mice. (a, c, and e) represent the expression of Aβ in DG, CA1, and CA3 for the higher‐dose lipopolysaccharide (H‐LPS) group; (b, d, and f) represent the expression of Aβ in DG, CA1, and CA3 for the control (CON) group, respectively; each image under 200× magnification

**Figure 6:**
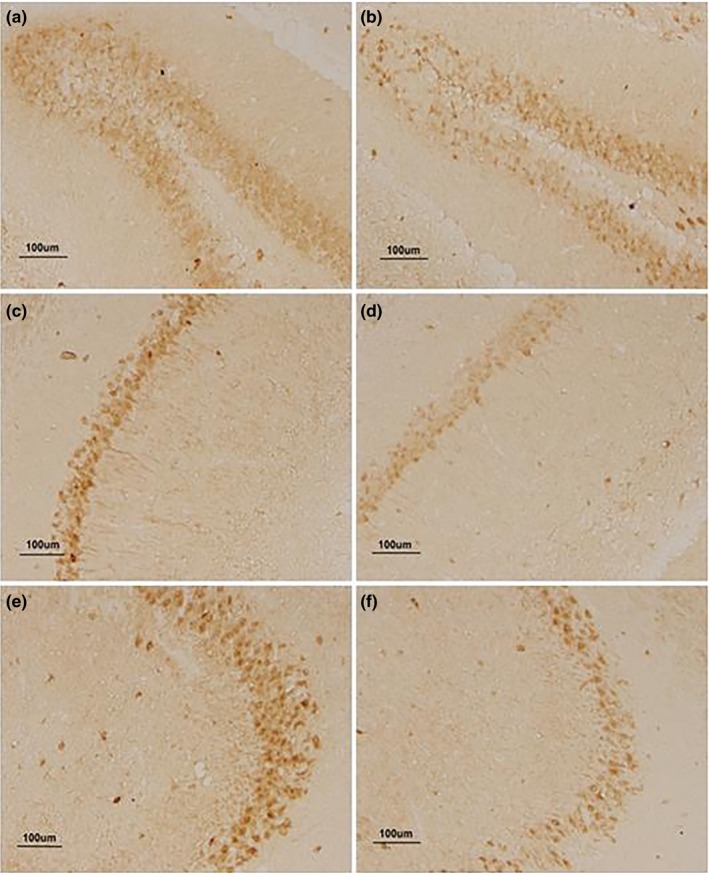
The expression of p‐tau in the hippocampus of 22‐month CD‐1 mice. (a, c, and e) represent the expression of p‐tau in DG, CA1, and CA3 for the higher‐dose lipopolysaccharide (H‐LPS) group; (b, d, and f) represent the expression of tau in DG, CA1, and CA3 for the control (CON) group, respectively; each image under 200× magnification

**Figure 7:**
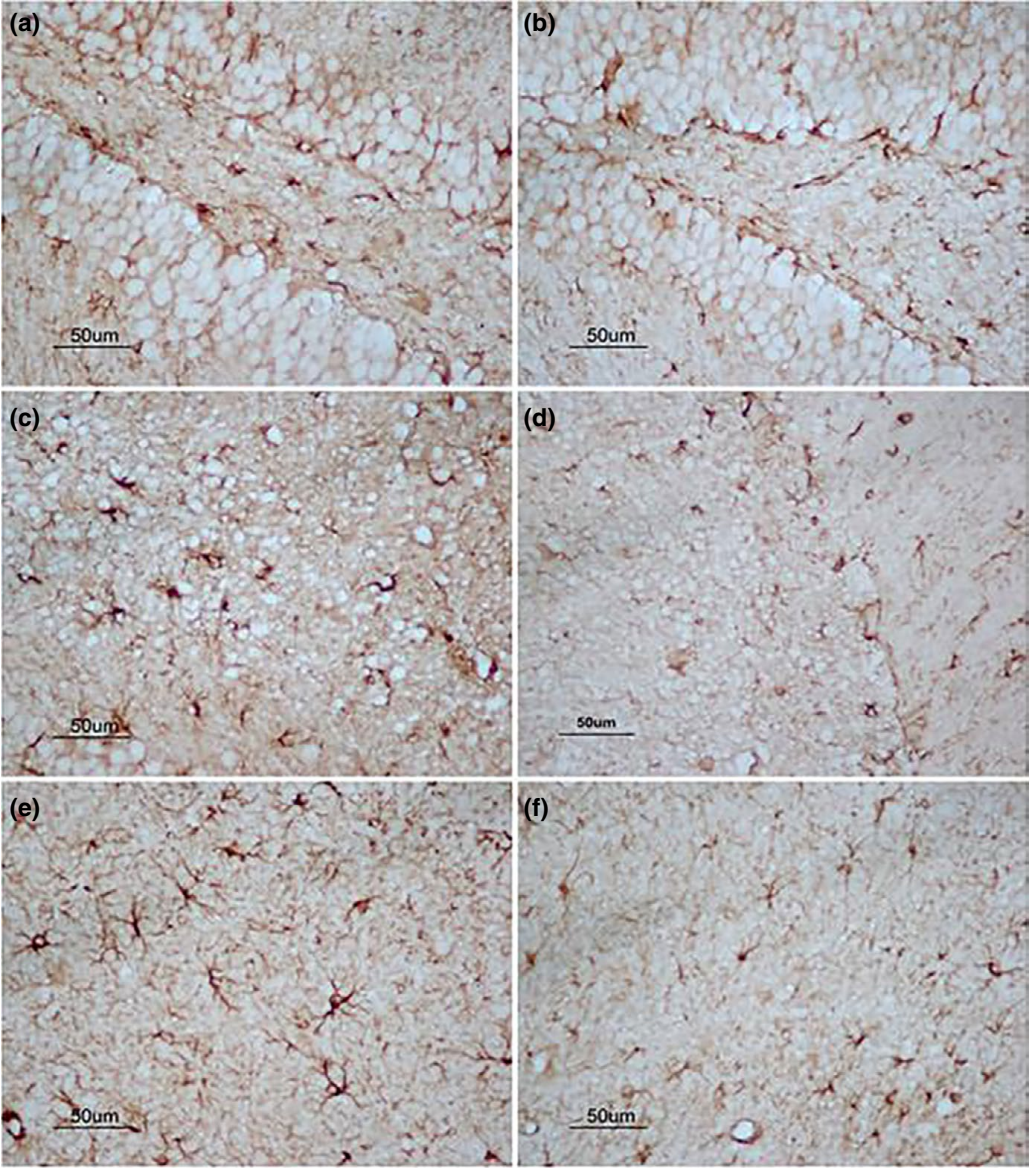
The expression of GFAP in hippocampus of 22‐month CD‐1 mice. (a, c, and e) represent the expression of GFAP in DG, CA1, and CA3 for the higher‐dose lipopolysaccharide (H‐LPS) group; (b, d, and f) represent the expression of GFAP in DG, CA1, and CA3 for the control (CON) group, respectively; each image under 400× magnification

For the 22 months old mice, the significantly increased levels of Aβ_42_, p‐tau, and GFAP were also found in most hippocampal layers (Tables 4, 5, Figures 5, 6, 7); that is, Aβ_42_ levels in DG‐GL, DG‐HL, DG‐MS, CA1‐RS, CA3‐LS, CA3‐PL, and CA3‐OS (*p*s < .05); p‐tau levels in DG‐MS, CA1‐MS, CA1‐RS, CA1‐PL, CA1‐OS, and CA3‐LS (*p*s < .05); and GFAP levels in DG, CA1, and CA3 (*p*s < .05). Compared to the CON group, the H‐LPS group showed higher levels of Aβ_42_ in almost hippocampal layers except CA1‐OS; the same was observed for L‐LPS in DG‐HL, DG‐MS, CA1‐RS, CA3‐LS, and CA3‐OS (*p*s < .05). Moreover, H‐LPS mice had higher Aβ_42_ levels in DG‐GL, DG‐HL, and CA3‐PL than L‐LPS mice (*p*s < .05). When the sex was separated, the H‐LPS males had higher levels of Aβ_42_ in DG‐GL, DG‐HL, DG‐MS, CA1‐PL, CA3‐LS, CA3‐PL, and CA3‐OS than the same‐sex CON (*p*s < .05), and higher levels of Aβ_42_ in CA3‐PL than the L‐LPS males (*p* = .023). Compared to the female CON, the H‐LPS female mice had significantly increased Aβ_42_ levels in DG‐HL, DG‐S, CA3‐MS, CA3‐LS, and CA3‐PL. In addition, the H‐LPS females had higher Aβ_42_ levels in DG‐HL, DG‐GL, and CA3‐PL than the L‐LPS females (*p*s < .05). H‐LPS mice held higher levels of p‐tau in almost subregion layers except for CA3‐OS than CON ones (*p*s < .05). L‐LPS mice had higher p‐tau levels in DG‐MS, CA1‐RS, and CA1‐OS than CON mice (*p*s < .05), but lower p‐tau levels in CA1‐PL and CA3‐LS than H‐LPS mice (*p*s < .05). When the sex was separated, the H‐LPS males had significantly increased p‐tau levels in DG‐MS, CA1‐PL, CA1‐OS, CA3‐MS, CA3‐LS, and CA3‐PL relative to the CON males (*p*s < .05) and increased p‐tau levels in CA3‐LS relative to the L‐LPS males (*p* = .041). The H‐LPS females had higher levels of p‐tau in DG‐MS, CA1‐MS, CA1‐PL, CA1‐OS, and CA3‐LS than the same‐sex CON (*p*s < .05) and higher levels of p‐tau in CA1‐MS than the L‐LPS females (*p* = .045). Meanwhile, the L‐LPS females had higher levels of p‐tau in CA1‐OS than the CON females (*p* = .037). H‐LPS mice showed higher levels of GFAP in DG, CA1, and CA3 than the CON ones (*p*s < .05) and higher levels of GFAP in CA1 than L‐LPS mice (*p* = .028). L‐LPS mice had higher levels of GFAP in CA3 than the CON mice (*p* = .031). The same was observed for the males in different groups. Compared to the CON females, the H‐LPS female mice had increased levels of GFAP in CA1 and CA3 (*p* = .029, .038), and so did the L‐LPS female mice in CA1 (*p* = .044).

### 3.8 Correlations between performances of RAWM and Aβ_42_, p‐tau, and GFAP proteins

Due to insignificant differences in the performances of RAWM among the three groups atone and 6 months old, the correlations were not analyzed between the performances of RAWM and the levels of Aβ_42_, p‐tau, and GFAP in different layers of the hippocampus in those ages. Table 6 displayed Pearson's correlation coefficients between the hippocampal protein levels, indicated as the mean in all subregion layers, and the RAWM performances for 12‐, 18‐ and 22‐month‐old mice.

**Table 6:** The correlations between spatial performances and Aβ, p‐tau, and GFAP levels in different sublayer of hippocampus

Protein	Ages	Layers	Groups	Learning phase		Memory phase	
				Number of errors (*P*)	Latency (*P*)	Number of errors (*P*)	Latency (*P*)
Aβ	12‐month	DG‐HL	All mice	0.467 (0.011)*	0.441 (0.016)*	0.394 (0.092)	0.427 (0.045)*
			H‐LPS	0.554 (0.048)*	0.553 (0.067)	0.284 (0.567)	0.325 (0.482)
		DG‐MS	All mice	0.192 (0.214)	0.317 (0.038)*	0.182 (0.244)	0.221 (0.154)
			H‐LPS	0.328 (0.256)	0.265 (0.373)	0.083 (0.778)	0.123 (0.675)
	18‐month	DG‐HL	All mice	0.451 (0.006)*	0.538 (0.004)*	0.320 (0.045)*	0.377 (0.018)*
			H‐LPS	0.041 (0.807)	0.072 (0.663)	0.067 (0.827)	0.219 (0.472)
		CA1‐RS	All mice	0.234 (0.146)	0.326 (0.047)*	0.059 (0.706)	0.139 (0.575)
			H‐LPS	0.062 (0.848)	0.081 (0.799)	0.482 (0.041)*	0.341 (0.234)
		CA1‐PL	All mice	0.318 (0.040)*	0.261 (0.096)	0.370 (0.021)*	0.473 (0.003)*
			H‐LPS	0.626 (0.017)*	0.625 (0.018)*	0.119 (0.685)	0.286 (0.339)
	22‐month	DG‐HL	All mice	0.375 (0.019)*	0.463 (0.003)*	0.370 (0.021)*	0.470 (0.003)*
			H‐LPS	0.486 (0.049)*	0.542 (0.027)*	0.119 (0.685)	0.276 (0.339)
		DG‐MS	All mice	0.501 (0.015)*	0.507 (0.014)*	0.274 (0.206)	0.415 (0.049)*
			H‐LPS	0.372 (0.086)	0.474 (0.028)*	0.020 (0.949)	0.278 (0.357)
			L‐LPS	0.063 (0.905)	0.175 (0.740)	0.124 (0.815)	0.446 (0.031)*
		CA1‐RS	All mice	0.327 (0.044)*	0.254 (0.104)	0.334 (0.036)*	0.306 (0.049)*
			H‐LPS	0.189 (0.556)	0.156 (0.627)	0.462 (0.049)*	0.082 (0.799)
		CA3‐PL	All mice	0.368 (0.015)*	0.411 (0.006)*	0.385 (0.011)*	0.365 (0.016)*
			H‐LPS	0.027 (0.929)	0.021 (0.945)	0.162 (0.581)	0.142 (0.643)
p‐tau	12‐month	DG‐MS	All mice	0.388 (0.012)*	0.495 (0.001)*	0.310 (0.055)	0.377 (0.018) *
			H‐LPS	0.488 (0.077)	0.631 (0.015)*	0.342 (0.277)	0.249 (0.434)
	18‐month	DG‐MS	All mice	0.231 (0.136)	0.393 (0.007)*	0.320 (0.045)*	0.398 (0.005)*
			H‐LPS	0.202 (0.507)	0.249 (0.412)	0.147 (0.631)	0.239 (0.432)
		CA1‐RS	All mice	0.016 (0.935)	0.123 (0.524)	0.063 (0.716)	0.069 (0.722)
			H‐LPS	0.676 (0.018)*	0.137 (0.653)	0.263 (0.363)	0.154 (0.602)
			L‐LPS	0.514 (0.047)*	0.024 (0.955)	0.405 (0.320)	0.179 (0.685)
	22‐month	DG‐MS	All mice	0.338 (0.027)*	0.370 (0.015)*	0.365 (0.036)*	0.285 (0.064)
			H‐LPS	0.016 (0.960)	0.453 (0.046)*	0.404 (0.029)*	0.090 (0.770)
		CA1‐RS	All mice	0.435 (0.022)*	0.426 (0.052)	0.041 (0.839)	0.413 (0.012)*
			H‐LPS	0.613 (0.050)	0.619 (0.031)*	0.387 (0.192)	0.325 (0.135)
			L‐LPS	0.569 (0.042)*	0.456 (0.017)*	0.348 (0.499)	0.348 (0.499)
		CA1‐OS	All mice	0.406 (0.068)	0.492 (0.035)*	0.473 (0.012)*	0.533 (0.065)
			H‐LPS	0.467 (0.098)	0.556 (0.049)*	0.214 (0.701)	0.138 (0.769)
			L‐LPS	0.316 (0.042)*	0.304 (0.559)	0.518 (0.036)*	0.327 (0.527)
		CA3‐PL	All mice	0.170 (0.301)	0.234 (0.151)	0.375 (0.020)*	0.352 (0.028)*
			H‐LPS	0.320 (0.265)	0.219 (0.452)	0.484 (0.076)	0.464 (0.048)*
GFAP	12‐month	CA3	All mice	0.393 (0.102)	0.423 (0.041)*	0.447 (0.416)	0.429 (0.311)
			H‐LPS	0.091 (0.718)	0.065 (0.639)	0.453 (0.259)	0.321 (0.232)
	18‐month	CA1	All mice	0.348 (0.116)	0.362 (0.045)*	0.254 (0.104)	0.227 (0.149)
			H‐LPS	0.266 (0.748)	0.281 (0.819)	0.156 (0.627)	0.189 (0.556)
		CA3	All mice	0.561 (0.019)*	0.552 (0.023)*	0.232 (0.400)	0.282 (0.244)
			H‐LPS	0.607 (0.041)*	0.621 (0.112)	0.162 (0.834)	0.113 (0.965)
	22‐month	DG	All mice	0.405 (0.064)	0.492 (0.036)*	0.483 (0.032)*	0.543 (0.095)
			H‐LPS	0.487 (0.095)	0.556 (0.089)	0.234 (0.701)	0.238 (0.766)
		CA1	All mice	0.368 (0.015)*	0.411 (0.026)*	0.385 (0.091)	0.365 (0.106)
			H‐LPS	0.027 (0.929)	0.021 (0.945)	0.265 (0.128)	0.142 (0.643)
		CA3	All mice	0.397 (0.031)*	0.376 (0.029)*	0.475 (0.351)	0.395 (0.043)*
			H‐LPS	0.432 (0.016)*	0.464 (0.017)*	0.196 (0.961)	0.324 (0.547)

Abbreviations: H‐LPS, higher‐dose LPS; L‐LPS, lower‐dose LPS; LPS, lipopolysaccharide.

* *p* < .05.

At the age of 12 months, positive correlations were significantly found between the number of errors and latency in the learning phase and the Aβ_42_ level in DG‐HL (*r* = .467, .441; *p*s < .05), and between the latency in the learning phase and the Aβ_42_ level in DG‐HL (*r* = .427, *p* = .045) for all mice combined. When the groups were separated, only the number of errors in the learning phase in the H‐LPS group significantly correlated with the Aβ_42_ level in DG‐HL (*r* = .554; *p* = .048). Significantly positive correlations were found between the learning errors and latency (*r* = .388, .495; *p*s < .05), the memory latency (*r* = .337; *p* = .018), and p‐tau level in the DG‐MS for all mice combined. Only the learning latency in the H‐LPS group significantly correlated with the p‐tau level in DG‐MS (*r* = .631; *p* = .015). The GFAP level positively correlated with only the latency in the learning phase (*r* = .423; *p* = .041).

At 18 months of age, there were significantly positive correlations between the errors and latency in the learning or memory phase and the Aβ_42_ level in DG‐HL for all mice combined (*p*s < .05), between the latency in the learning phase and Aβ_42_ level in CA1‐RS (*r* = .326; *p* = .047), and between the learning‐phase errors, memory‐phase errors and latency, and the Aβ_42_ level in CA1‐PL (*p*s < .05). For each group, H‐LPS mice had significantly positive correlations between the memory‐phase errors and the Aβ_42_ level in CA1‐RS (*r* = .482, *p* = .041), and between the learning‐phase errors and latency and the Aβ_42_ level in CA1‐PL (*r* = .626, .625; *p*s < .05). For all mice combined, positive correlations were found between the learning‐phase latency and p‐tau level in DG‐MS (*r* = .393; *p* = .007) and between the memory‐phase errors and latency and p‐tau level in DG‐MS (*r* = .320, .398; *p*s < .05). When the groups were separated, the learning‐phase errors in both H‐LPS and L‐LPS mice significantly correlated with the p‐tau level in CA1‐RS (*r* = .676, .516; *p*s < 0.05). The GFAP level in CA1 positively correlated with the latency in the learning phase (*r* = .362, *p* = .045), and so were GFAP level in CA3 with the errors and latency in the learning phase for all mice combined (*r* = .561, .552; *p*s < .05). Only the learning errors in H‐LPS mice significantly correlated with the GFAP level in CA3 (*r* = .607, *p* = .041).

For 22‐month‐old mice, positive correlations occurred between the errors and latency in both the learning and the memory phases and the Aβ_42_ level in DG‐HL and CA3‐PL (*p*s < .05), and between the learning errors or memory errors and latency and CA1‐RS Aβ_42_ (*p*s < .05). For each group, H‐LPS mice showed positive correlations between memory errors and latency and DG‐HL Aβ_42_ (*r* = .486, .542; *p*s < .05), between learning latency and the Aβ_42_ level in DG‐MS (*r* = .474, *p* = .028), and between memory errors and the Aβ_42_ level in CA1‐RS (*r* = .462, *p* = .049). In addition, L‐LPS mice had positive correlations only between memory latency and DG‐MS Aβ_42_ (*r* = .446, *p* = .031). For all mice combined, there were significantly positive correlations between the learning errors and latency or memory errors and the p‐tau level in DG‐MS (*p*s < .05), between the learning errors and memory latency and the p‐tau level in CA1‐RS (*r* = .435, .413; *p*s < .05), between the learning latency and memory errors and the p‐tau level in CA1‐OS (*r* = .492, .473; *p*s < .05), and between the memory errors or latency and the p‐tau level in CA3‐PL (*r* = .375, .352; *p*s < .05). For each group, H‐LPS mice had positive correlations between learning latency and memory errors and the p‐tau level in DG‐MS (*r* = .453, .404; *p*s < .05), between learning latency and the p‐tau level in CA1‐RS and CA1‐OS (*r* = .619, .556; *p*s < .05), and between memory latency and the p‐tau level in CA3‐PL (*r* = .464, *p* = .048). L‐LPS mice had positive correlations between the errors and latency in the learning phase and the p‐tau level in CA1‐RS (*r* = .569, .456; *p*s < .05), and between the errors in two phases and the p‐tau level in CA1‐OS (*r* = .316, 0.518; *p*s < .05). Significantly positive correlations were found between learning latency and memory errors and GFAP levels in DG (*r* = .492, .483; *p*s < .05), and between the errors and latency in the learning phase and GFAP levels in CA1 and CA3 for all mice combined (*p*s < .05). For each group, only the learning errors and latency in H‐LPS group significantly correlated with the GFAP level in CA3 (*r* = .432, .464; *p*s < .05). In conclusion, these correlations were found in more layers of the hippocampus as the age increased (18 and 22 months).

## 4 DISCUSSION

### 4.1 LPS exposure during late embryogenesis aggravated the age‐related changes of behaviors in CD‐1 mice

Aging is a normal physiological process and commonly correlated with malfunction in many domains of brain function, such as motor and cognition. It is also a major risk factor of many neurodegenerative diseases, such as AD and Parkinson's disease. The neurobiological mechanisms underlying aging and AD may share some common pathogenesis, such as chronic inflammation and oxidative stress. The state of pregnancy is vulnerable to bacteria or viral infections, such as urinary, respiratory tract, enteric, and periodontal infections, which can result in behavioral, morphological, and immunological changes in the offspring (Dinel et al., 2014). Moreover, infections at different periods of prenatal development may have diverse neurodevelopmental consequences. Infections in late embryogenesis have a detrimental and long‐term effect on cognitive function during adulthood and aging (Meyer et al., 2006), indicating a causal relationship between disturbances of late embryonic development and the risk of AD‐like neuropathology (Krstic et al., 2012). LPS is the main component of the outer membrane of gram‐negative bacteria, and systemic LPS injections trigger neuroinflammation (Wang et al., 2017). Exposure to LPS in early gestation is related to fetal death and resorption, but exposure to LPS in mid‐ to late gestation is associated with fetal death and preterm delivery. In the present study, the pregnant CD‐1 mothers were i.p. given 50 or 25 μg/kg of LPS every day during late gestation (gd 15–17) to simulate prenatal inflammation, and we further examined its long‐term effect on age‐related behavioral changes in their offspring. In this experiment, we randomly extracted some mice from their litters at each age to examine their behaviors to avoid the effects of repetitive measurement on their behaviors and dynamically detected their body weight 21 days–22 months of age. Fetal death and preterm delivery were not observed here, which may be due to the use of relatively low doses of LPS. In addition, maternal LPS‐exposed and control CD‐1 mice at different ages had similar body weights, indicating that they experienced normal physical development and maturation.

Memory impairments, especially episodic memory, occur as a consequence of normal aging across many species, including humans and rodents. Aged mice in different strains have significantly reduced spatial learning and memory abilities, and the onset of this age effect begins at middle age (even early adulthood) and persistently progresses onwards, such as SAMP8 and Kunming mice (Cao et al., 2013, 2012; Chen et al., 2007; Currais et al., 2018). Bacterial infections during pregnancy result in a systemic inflammatory reaction in mothers and can affect cognition in their offspring. Our recent findings suggested that age significantly affected spatial learning and memory from middle age to old age in CD‐1 mice, and maternal exposure to LPS in CD‐1 mice could trigger and exacerbate the age‐related spatial memory impairment in their offspring from middle age onwards in a linear manner (Li, Cao, et al., 2016; Li, Wang, et al., 2016). In the current study, we used the same system to mimic maternal systemic inflammation during pregnancy and examined its long‐term effect on recognition and spatial learning and memory in their offspring. The OLR task is employed to assess recognition memory, which is hippocampus‐dependent. Although this task has been used as a tool to evaluate the effects of age on memory and recognition, direct evidence for whether exposure to LPS induces impairments in the OLR task is lacking. In this study, the LPS‐exposed mice at ages of 1 and 6 months had similar recognition memory in the OLR task. However, H‐LPS offspring at 12 months showed a reduced PI_10 min_ for novel object location in a 10‐min delay test compared to control mice of the same age, which was attributable to the females. This damage effect continued to strengthen until the senectitude. At 18 and 22 months old, the H‐LPS group had significantly lower PIs for novel object location in 10‐min and 24‐hr delay tests than the same‐age controls, which was also contributable to the females. Moreover, L‐LPS at ages of 18 and 22 months showed a lower PI_10 min_ for novel object location in the 10‐min phase than the same‐age controls. Previous research has indicated that adult offspring suffered with LPS in the embryonic stage showed recognition memory impairments in the novel object recognition task (Wischhof et al., 2015). Our results also indicate that maternal inflammatory insult during pregnancy could impair recognition memory in offspring mice; moreover, this impairment began in midlife and persistently progressed onwards. In addition, we also found that the damage of recognition memory in the 10‐min phase emerged earlier than in the 24‐hr phase, and the LPS treatment effect displayed a significant dose‐related pattern. For instance, the damage effect of the H‐LPS group was clearer and occurred earlier than that of the L‐LPS group. Due to being more sensitive than the Morris water maze, the RAWM task is used to evaluate spatial learning and memory in this study, which is also hippocampus‐dependent (Chen, Wang, Wang, & Zhou, 2004; Yang, Chen, Wang, & Wang, 2015). It was found that maternal LPS exposure could trigger and aggravate the age‐related impairments in spatial learning and memory in their offspring from middle age onward in a linear manner, and this LPS treatment effect displayed a significant dose‐related pattern, consistent with our recent finding (Li, Cao, et al., 2016; Li, Wang, et al., 2016).

Strong evidence indicates that LPS exposure during late embryogenesis could also result in noncognitive behavioral abnormalities in pre‐ or adult, such as altered anxiety‐like and depressive‐like behaviors and locomotor activity, prepulse inhibition deficits, and impaired species‐typical behaviors (hoarding and nesting; Asiaei, Solati, & Salari, 2011; Enayati et al., 2012; Fortier et al., 2007; Glass et al., 2019; Golan, Stilman, Lev, & Huleihel, 2006; Hsueh et al., 2017; Penteado et al., 2014; Wischhof et al., 2015). But it is noteworthy that these studies varied with respect to different methodology used, making it difficult to compare across studies. In the current study, the LPS‐exposed mice at ages of 1 and 6 months showed similar species‐typical behavior in the nesting task, sensorimotor ability in the beam walking task, locomotor activity in the open field task, and anxieties in the open field and elevated plus maze tasks compared to the CON mice, suggesting that they experienced normal maturity of the central nervous system in adolescence and adulthood. However, up to 18 and 22 months, H‐LPS mice showed reduced scores in the nesting task and balance times in the beam walking task, and only 22‐month‐old L‐LPS mice showed similar changes. Relative to the same‐age controls, H‐LPS mice at ages of 12–22 months had a small number of squares crossed in the open field and open arms times in the elevated plus maze. However, these LPS effects on locomotor activity and anxiety‐like behavior were not observed in the L‐LPS mice. These findings suggested that prenatal exposure to low doses of LPS exhibited decreased species‐typical behavior, sensorimotor ability, and locomotor activity, and increased anxiety from middle age onward, which also showed a significant dose‐related pattern. These behavioral changes in the middle‐aged mice were consistent with the results from our previous study (Chen et al., 2011). Interestingly, we also found that the females' damage emerged earlier than the males' as some of the treatment effect in 12‐month‐old mice only occurred in the females. These findings indicated the females were more vulnerable to this inflammatory insult than the males. However, another study found that the males were more severely influenced than the females in the object recognition memory decline induced by LPS administration (Wischhof et al., 2015), which seemed inconsistent with our data. It appears that this discrepancy is caused by different detection methods, and OLR rather than object recognition was assessed in this study.

In sum, the offspring mice, whose mothers were exposed to low doses of LPS during late pregnancy, could experience normal development and maturity of the central nervous system in adolescence and adulthood, but had more significantly accelerated age‐related behavioral changes in middle and old age, which seems consistent with the behavioral changes in AD.

### 4.2 LPS exposure during late pregnancy accelerated age‐related changes of Aβ and p‐tau

Senile plaques accumulate in extracellular spaces as a result of the gradual deposition and accumulation of specific Aβ peptides. The length of Aβ varies, but a 42‐amino acid variant (Aβ_42_) is regarded neurotoxic because of its propensity to readily aggregate into oligomers and fibrils (Zhang, Thompson, Zhang, & Xu, 2011). The insoluble Aβ_42_ progressively increases with age and further aggravates in the AD brain (Zheng & Koo, 2011). Tau is a microtubule‐associated protein, and p‐tau leads it to disconnect from the microtubules and accumulate within the axoplasm as neurofibrillary tangles (Iqbal et al., 2005). Furthermore, tau dissociation causes a reduction in microtubule stability and impaired axonal transport, ultimately resulting in neuronal malfunction and the loss of synapses and subsequent retrograde degeneration (Iqbal & Grundke‐Iqbal, 2005). Increasing evidence indicates that Aβ oligomer trigger neurotoxicity, likely via p‐tau (Bennett et al., 2017; Selenica et al., 2013). Cognitive impairment in AD occurs before the appearance of amyloid plaques and neurofibrillary tangles, although the soluble Aβ oligomers and hyperphosphorylated tau damage cognitive function (Lesne et al., 2006; Pater, 2011). Many studies have highlighted chronic neuroinflammation as a dedicator to the pathogenesis of AD (Cole et al., 2017; Mesquita et al., 2016). For instance, neuroinflammation induced by the repeated administration of LPS led to an accumulation of Aβ_42_ in the hippocampus and cerebral cortex of an outbred ICR mouse (Lee et al., 2008). Repeated peripheral injections of LPS generated both an increase in Aβ_42_ peptide and the presence of plaques in the hippocampus of C57BL/6J mice (Kahn et al., 2012). Infection‐induced chronic inflammation significantly aggravates tau pathological characteristics in a3xTg‐AD mouse model (Sy et al., 2011). However, the evidence that prenatal chronic infection affects the expression of Aβ and tau in the brain is sparse and limited. Only one study indicated that pregnant rats were intraperitoneally injected with LPS (0.4 mg/kg), and their pups at the age of 3 months old had an increased expression of tau in the hippocampus (Wang et al., 2018).

In our study, although we did not observe senile plaques and neurofibrillary tangles in each subregion of the hippocampus in LPS‐challenged mice, the changes in Aβ_42_ and p‐tau observed in the prenatally challenged mice from middle age onward were consistent with AD‐related pathology. The LPS‐exposed mice displayed an increase in the intensity of immunoreactivity for Aβ_42_ and p‐tau in the hippocampus, and this LPS‐treated effect showed a significant age‐ and dose‐related pattern. For instance, the H‐LPS group had significantly elevated Aβ_42_ and p‐tau only in some layers of the hippocampal subregions than the same‐age CON group in the 12‐month‐old mice, which was attributable to the females. But, compared to the same‐aged controls, H‐LPS mice at ages of 18 and 22 months showed significantly increased Aβ_42_ and p‐tau in most layers of the hippocampal subregions. In the 18‐ and 22‐month‐old mice, the L‐LPS group exhibited significantly elevated Aβ_42_ and p‐tau only in some layers of the hippocampal subregions in comparison with the same‐age CON. These results were in accordance with the performances in the RAWM. The correlation analysis indicated that the changed Aβ_42_ and p‐tau levels significantly correlated with impaired spatial learning and memory abilities in the RAWM. It is worth noting that this correlation also showed dose‐related and age‐dependent effects. For example, the correlations between the Aβ_42_ level and the errors and latency in two RAWM phases only positively existed in DG‐HL at 12 months old, but it also existed in CA1‐PL and CA1‐RS at 18 months old with the addition of CA3‐PL and DG‐MS at 22 months old. Meanwhile, a positive correlation between the p‐tau level and the errors and latency only existed in DG‐MS at 12 and 18 months old for all mice combined; this also existed in CA1‐RS, CA1‐OS, and CA3‐PL at 22 months old. These correlations above were almost observed in the H‐LPS group at the ages of 12, 18, and 22 months, indicating an LPS treatment effect; these correlations were observed in the L‐LPS group at the age of 18 months between the CA1‐RL p‐tau level and learning latency, and also at the age of 22 months between the p‐tau level in CA1‐RS and CA1‐OS and the errors and latency, indicating an LPS‐dose effect. Collectively, these observations support the hypothesis that inflammatory exposure during late embryogenesis can trigger and exacerbate the changes of the hippocampal Aβ_42_ and p‐tau levels from middle age onward.

### 4.3 LPS exposure during late pregnancy aggravated age‐related change of GFAP

Neuroinflammation is involved in the development of aging and amyloid plaques in AD. Astrocytes and microglia are fundamental in defending the brain against infection and inflammation. With increasing age, microglia and astrocytes, the two major cell effectors, contribute to the chronic activation of neuroinflammation as well as the overexpression of proinflammatory cytokines and reactive oxygen species (Meghraj et al., 2017). Increasing evidence suggests that astrocytosis and microglia are an early phenomenon involved in the synaptic function of adjacent neurons and reducing their neuroprotective activity; these mechanisms may be contributing to the important pathologic change in AD development (Barnes et al., 2015; Carter et al., 2012; Ramirez et al., 2017). GFAP is the main intermediate filament protein, which is considered as a specific marker of mature astrocytes (Hayakawa, Kato, & Araki, 2007). In humans and rodents, the expression of GFAP mRNA and protein with age gradually increases (Hayakawa et al., 2007; Salminen et al., 2011). The enlargement of the astrocytic body and increase in GFAP expression indicate reactive gliosis, a process highly associated with brain damage and aging (Bellaver, Souza, Souza, & Quincozes‐Santos, 2017). Prenatal exposure LPS resulted in a significant GFAP increase in the hippocampal CA1 region, and this condition continued from 3‐ to 20‐month‐old offspring rats (Hao et al., 2010).

In our experiment, LPS treatment significantly aggravated the increase of GFAP in different subregions of the hippocampus in comparison with the same‐age control groups from midlife onward. This effect started at 12 months and achieved the maximum at 22 months. For instance, the treatment effect on GFAP in the H‐LPS group at the age of 12 months was intensively enlarged to CA3. However, this treatment‐related difference seemed to be strengthened at 22 months old. At this age, L‐LPS had significantly higher GFAP only in CA3 relative to the same‐age CON. The correlation analysis showed that the changed GFAP level significantly correlated with the impairment of spatial learning and memory abilities in the RAWM. It is noticeable that these correlations also showed dose‐related and age‐dependent effects. For example, the correlation between the GFAP level and the errors and latency in two RAWM phases only positively existed in CA3 at 12 months old, but it also occurred in CA1 at 18 months old with the addition of DG at 22 months old. Only the H‐LPS group at the ages of 18 and 22 months showed positive correlations between the changed GFAP level and the errors and latency, indicating LPS treatment and LPS‐dose effects. This indicates that adverse pregnancy may lead to a trend prone to form AD‐related pathology in middle‐ and older‐aged mice.

In sum, intrauterine inflammation induced by LPS significantly impacts late‐life behavioral performance and neuropathology in CD‐1 mice. Furthermore, these LPS effects displayed a significant dose‐related pattern and some differences between sexes. For instance, the damage effect of L‐LPS occurred earlier and was more obvious than that of L‐LPS. The females were more vulnerable to this inflammation insult induced by LPS than the males. The possibilities of these differences are the severity of inflammation suffered in the late embryo stage and different estrogen levels in mice.

## 5 CONCLUSIONS

Maternal inflammatory insult by LPS administration during pregnancy revealed a significant augmentation of age‐related behavioral changes in CD‐1 mice, including decreased nesting and sensorimotor abilities, increased anxiety, and reduced recognition memory and spatial learning and memory. The latter was associated with Aβ_42_ load and p‐tau level elevations and hyper‐activity of astrocytes in the dorsal hippocampus. The changes in behavior and hippocampal pathology in these mice seemed fairly consistent with the changes in AD. Although this study is limited by only using non‐transgenic CD‐1 mice and the imprecise semi‐quantitative method of immunohistochemistry, it did reveal a possibility that inflammation exposure during pregnancy could contribute to AD neuropathology and exacerbate the course of the disease, but the precise mechanism of this notion requires further research.

## CONFLICT OF INTEREST

The authors declare no competing interest.

## AUTHORS' CONTRIBUTION

FW conceived of the study, carried out behavioral tasks and drafted the manuscript. ZZ participated in immunohistochemical test and conducted the statistical analysis. LC, QY, and QL participated in behavioral tasks and the immunohistochemical test. GC conceived the study, and took part in its design and organized and helped to draft the manuscript. Each author read and approved the final manuscript.

## ETHICAL APPROVAL AND CONSENT TO PARTICIPATE

This study was carried out in accordance with the recommendations of the National Institutes of Health (NIH) Guide for the Care and Use of Laboratory Animals and the Center for Laboratory Animal Sciences at Anhui Medical University. The protocol was approved by the “Laboratory Animal Welfare & Ethics committee.”

## Data Availability

The data that support the findings of this study are available from the corresponding author upon reasonable request.
