# Supplementary material for: Lipopolysaccharide exposure during late embryogenesis triggers and drives Alzheimer‐like behavioral and neuropathological changes in CD‐1 mice
Source: Brain Behav. 2020 Jan 30;10(3):e01546. doi: 10.1002/brb3.1546 (PMC7066339; doi:10.1002/brb3.1546)
Supplement: Supplementary file 1 [file BRB3-10-e01546-s001.doc]

Supplementary Fig 1 The body weights (g) of CD-1 mice with different treatment in 1 month (A and B) and 6 month (C and D)

Supplementary Fig 2 The performance of 1-month CD-1 offspring with different exposure in the learning (A-D) and memory (E-H) phases of RAWM.

Supplementary Fig 3 The performance of 6-month CD-1 offspring with different exposure in the learning (A-D) and memory (E-H) phases of RAWM.

Supplementary table 1 The behavioral results of different treated CD-1 mice at the age of 1 and 6 months

| Tasks | index | Ages | H-LPS group | | | L-LPS group | | | Controls | | |
| --- | --- | --- | --- | --- | --- | --- | --- | --- | --- | --- | --- |
|  |  |  | All mice (n=16) | Males  (n=8) | Females  (n=8) | All mice  (n=16) | Males  (n=8) | Females  (n=8) | All mice  (n=16) | Males  (n=8) | Females  (n=8) |
| Nesting | scores | 1-month | 2.0(0/3.0) | 1.5(0/2.25) | 3.0(0/3.0) | 2.0(0/3.0) | 1.0(0/3.0) | 2.5(0.75/3.0) | 2.0(0/3.0) | 2.0(0.75/3.0) | 1.0(0/3.0) |
|  |  | 6-month | 2.5(2.0/3.0) | 3.0(2.25/3.75) | 2.0(1.25/2.75) | 3.0(2.0/3.0) | 2.5(2.0/3.0) | 3.0(2.0/3.0) | 3.0(2.0/3.0) | 2.5(2.0/3.0) | 3.0(2.25/3.0) |
| Beam walking | time (s) | 1-month | 60.0  (53.7/60.0) | 60  (48.1/60.0) | 60.0  (55.8/60.0) | 60  (46.3/60.0) | 60.0  (43.5/60.0) | 60.0  (51.2.0/60.0) | 60.0  (55.5/60.0) | 60.0  (53.0/60.0) | 60.0  (60.0/60.0) |
|  |  | 6-month | 60.0  (55.25/60.0) | 60  (54.25/60.0) | 60.0  (54.75/60.0) | 60  (60.0/60.0) | 60.0  (58.5/60.0) | 60.0  (60.0/60.0) | 60.0  (54.5/60.0) | 60.0  (52.0/60.0) | 60.0  (57.3/60.0) |
| Open field | peripheral time (s) | 1-month | 262.9±4.52 | 264.8±6.39 | 260.9±6.39 | 266.9±4.52 | 271.0±6.39 | 257.9±6.39 | 271.8±4.52 | 273.1±6.39 | 267.2±6.39 |
|  |  | 6-month | 259.1±5.58 | 259.5±7.89 | 258.8±7.89 | 248.5±5.58 | 241.1±7.89 | 258.9±7.89 | 261.2±5.58 | 261.4±7.89 | 261.0±7.89 |
|  | squares crossed | 1-month | 144.6±6.84 | 151.6±11.85 | 140.7±11.85 | 141.9±6.84 | 146.6±11.85 | 139.3±11.85 | 154.7±6.84 | 158.7±11.85 | 149.6±11.85 |
|  |  | 6-month | 177.9±8.39 | 178.3±11.70 | 177.6±11.70 | 168.3±8.39 | 175.5±11.70 | 161.8±11.70 | 152.5±8.39 | 148.5±11.70 | 156.5±11.70 |
| Elevated plus maze | number of entries | 1-month | 0.0  (0.0/1.75) | 0.0  (0.0/1.0) | 1.0  (0.0/3.25) | 1.0  (0.0/1.75) | 0.5  (0.0/1.0) | 1.0  (0.0/2.0) | 0.5  (0.0/2.0) | 0.5  (0.0/1.25) | 0.5  (0.0/3.0) |
|  |  | 6-month | 3.0  (1.0/5.0) | 3.0  (2.25/5.75) | 2.0  (0.25/5.0) | 2.0  (1.0/4.0) | 2.0  (0.25/5.0) | 2.0  (1.25/3.75) | 4.0  (1.25/4.0) | 4.0  (1.0/4.0) | 3.5  (1.25/4.0) |
|  | time (s) | 1-month | 15.6±4.59 | 18.9±6.49 | 13.3±6.49 | 10.8±4.59 | 9.3±6.49 | 12.2±6.49 | 14.4±4.59 | 12.6±6.49 | 17.4±6.49 |
|  |  | 6-month | 67.6±12.68 | 70.9±17.93 | 64.4±17.93 | 55.4±12.68 | 57.5±17.93 | 53.3±17.93 | 64.6±12.68 | 59.5±17.93 | 67.8±17.93 |
| object-location recognition | PI_10min_ | 6-month | 0.618±0.041 | 0.664±0.044 | 0.573±0.044 | 0.585±0.041 | 0.611±0.044 | 0.559±0.044 | 0.613±0.041 | 0.639±0.044 | 0.587±0.044 |
|  | PI_24h_ | 6-month | 0.545±0.038 | 0.474±0.035 | 0.616±0.035 | 0.570±0.038 | 0.523±0.035 | 0.618±0.035 | 0.533±0.038 | 0.486±0.035 | 0.580±0.035 |
